# Utility and variability of three non-invasive liver fibrosis imaging modalities to evaluate efficacy of GR-MD-02 in subjects with NASH and bridging fibrosis during a phase-2 randomized clinical trial

**DOI:** 10.1371/journal.pone.0203054

**Published:** 2018-09-07

**Authors:** Stephen A. Harrison, Andrea Dennis, Martine M. Fiore, Matt D. Kelly, Catherine J. Kelly, Angelo H. Paredes, Jennifer M. Whitehead, Stefan Neubauer, Peter G. Traber, Rajarshi Banerjee

**Affiliations:** 1 Division of Cardiovascular Medicine, Radcliffe Department of Medicine, University of Oxford, Oxford, United Kingdom; 2 Perspectum Diagnostics, Oxford, United Kingdom; 3 San Antonio Military Medical Center, San Antonio, Texas, United States of America; 4 Galectin Therapeutics Incorporated, Norcross, Georgia, United States of America; Kaohsiung Medical University, TAIWAN

## Abstract

**Background:**

Given the worldwide prevalence of NAFLD and NASH, there is a need to develop treatments to slow or reverse disease progression. GR-MD-02 (galactoarabino-rhamnogalaturonate) has been shown to reduce hepatic fibrosis in animal studies, and lower serum biomarkers of NASH fibrogenesis in humans. The primary aim of this study was to determine the difference between four-months of treatment with GR-MD-02 or placebo in liver inflammation and fibrosis as measured by iron-corrected T1 (cT1) mapping, a non-invasive magnetic resonance imaging (MRI) biomarker that correlates with the extent of hepatic fibro-inflammatory disease. The secondary aims were to determine change in liver stiffness as measured by magnetic resonance elastography (MRE) and shear-wave ultrasonic elastography (LSM), and to explore test-retest repeatability of the three biomarkers.

**Materials and methods:**

Thirty subjects (13 females, 46–71 years) with NASH and advanced fibrosis were recruited. Subjects were randomized to receive 8 mg.kg-1 GR-MD-02 (via IV infusion) or placebo, administered biweekly over a 16-week period. Therapeutic efficacy was examined using cT1, MRE, and LSM. Statistical analyses on group differences in the biomarkers were performed using robust ANCOVA models adjusting for baseline measurement and additional covariates.

**Results:**

There was no significant difference in cT1 (p = 0.16) between GR-MD-02 and placebo groups following a 16-week intervention. There was also no significant difference in liver stiffness, measured by MRE (p = 0.80) or LSM (p = 0.63), between groups. Examination of repeatability of the cT1, MRE and LSM revealed coefficient of variations of 3.1%, 11% and 40% respectively.

**Conclusions:**

8 mg.kg^-1^ of GR-MD-02 had no significant effect on non-invasive biomarkers of liver inflammation or fibrosis over a 4-month period. Histological confirmation was not available in this study. The high reproducibility of the primary outcome measure suggests that cT1 could be utilized for monitoring longitudinal change in patients with NASH.

## Introduction

Non-alcoholic fatty liver disease (NAFLD) represents a continuum of disease from simple steatosis (or fatty liver) to the more advanced non-alcoholic steatohepatitis (NASH). Estimates of the worldwide prevalence of NAFLD range from 6.3% to 33% with a median of 20% in the general population [1]; however, a study in asymptomatic middle-aged adults in the US suggests that this may be as high as 46% [2]. Simple steatosis is characterized by excessive fat accumulation in hepatocytes. The addition of inflammatory cell infiltrates, evidence of damage to hepatocytes (ballooning degeneration), and the deposition of fibrous tissue, are associated with the onset of NASH. Between 3 to 5% of Americans are estimated to have NASH [1], with as many as 12.2% found in asymptomatic adults [2]. While the prevalence of cirrhosis in NASH is not clearly defined, as many as one third of NASH patients progress to advanced fibrosis [3]. The only curative option available to these advanced patients is liver transplantation. Currently, the percentage of liver transplantations performed in the US for NASH is between 10% and 15%, but numbers are increasing, and NASH may become the leading cause for liver transplantation over the next 20 years [4].

The galectin-3 protein has recently been implicated in the pathogenesis of NASH. Galectins are a family of proteins with the property of binding avidly to galactose containing oligosaccharides associated with glycoproteins [5]. GR-MD-02 (galactoarabino-rhamnogalaturonate) represents a novel agent for the therapy of patients with NASH and advanced fibrosis. GR-MD-02 has been tested in two rodent models of liver fibrosis; a mouse model that reliably produced a pathological picture of NASH with fibrosis [6], and a toxin-induced model of liver fibrosis in rats [7]. Studies completed in the mouse NASH model showed GR-MD-02 reduced or eliminated fibrosis as measured by liver collagen content, and reduced the expression of galectin-3 in liver macrophages [6]. Much more robust fibrosis and cirrhosis were induced in rats treated with thioacetamide in which animals developed fibrosis that replaced 25% of the liver with collagen and had all the pathological characteristics of cirrhosis. Treatment of these cirrhotic rats with 4 weekly doses GR-MD-02 resulted in reduction of collagen to below 10%, reversal of cirrhosis, and reduced portal hypertension [7]. Furthermore, results from a phase 1 study in humans [8] indicated GR-MD-02 was safe and well tolerated at single and multiple intravenous infusions at doses of 2, 4 and 8 mg.kg-1, and pharmacokinetics showed drug exposure in humans at 8 mk.kg-1 was equivalent to the upper range of the targeted therapeutic dose determined from effective doses in NASH animal models. Additionally, there was evidence of an effect of GR-MD-02 on a relevant disease markers, with a dose-dependent reduction in serum biomarkers of NASH [9] due to a reduction in alpha-2 macroglobulin levels, and a trend that suggested reduced liver stiffness (measured with shear wave elastography), possibly related to a reduction in liver fibrotic tissue.

Based on the promising effects of GR-MD-02 seen in the animal models of NASH, and the positive phase 1 results, this study evaluated the efficacy of GR-MD-02 for the treatment of liver fibrosis in patients with NASH with advanced (bridging) fibrosis in a phase 2 clinical trial using non-invasive imaging modalities. Whilst liver biopsy remains the gold standard for evaluating liver pathology, its limitations (e.g. significant risk of serious complications, examination of only 0.002% of the liver, high intra- and inter-observer variability in histological interpretation) suggest there is a real clinical need for non-invasive tools to evaluate and monitor liver disease. Metrics derived from liver magnetic resonance imaging (MRI) are emerging as promising biomarkers due to being non-invasive, non-ionising and quantitative. MRI exploits the magnetic properties of hydrogen nuclei protons within a determined magnetic field. MRI-T1 mapping has shown promise as an effective non-invasive biomarker of fibroinflammatory disease in the liver [10–12], and also in other organs [12–14], as T1 relaxation time lengthens with increases in extracellular fluid (which may be caused by fibrosis and/or inflammation, as well as by elevated fat [11], often a precursor to pronounced hepatocyte injury [15]).The presence of iron however, which can be accurately measured from MRI-T2star (T2*), shortens the T1 [16], and thus must be accounted for. An algorithm has been created that allows for the bias introduced by elevated iron to be removed from the T1 measurements, yielding the iron corrected T1 [17,18]. The MRI-derived iron corrected T1 (cT1) has been shown to correlate with fibro-inflammatory disease [17,19] and can effectively stratify patients with NASH and cirrhosis [17]. Liver stiffness can also be measured using magnetic resonance elastography (MRE), and ultrasound-based transient elastography, both of which have been used to assess fibrosis and for predicting clinical outcomes [20–25].

The primary objective of this study was therefore to determine any difference in change in cT1 between subjects receiving either four-month treatment with GR-MD-02 or placebo. The secondary objectives were: (1) to determine the change in liver stiffness as measured by MR elastography (MRE) and shear-wave ultrasonic elastography (liver stiffness measurement; LSM) in the same groups; (2) measure change in liver iron and fat (derived from MRI-T2* and MRI-Proton Density Fat Fraction, PDFF); (3) and examine the test-retest repeatability of the three biomarkers of fibrosis.

The hypothesis was there would be a statistically significant reduction in the primary non-invasive biomarker of fibro-inflammatory disease (cT1) and in the secondary biomarkers of fibrosis (MRE and LSM) in the GR-MD-02 group compared to the placebo group.

## Materials and methods

### Study population

Thirty biopsy-confirmed NASH patients (13 Female, mean age 58.2 [range 46–71 years]) were recruited. A sample size of 30 subjects (15 in each group) was calculated based on the comparison of the primary efficacy variable, change in cT1 from baseline, with the following assumptions: (i) Difference in mean change in cT1 between treatment and placebo groups of 45.1 ms; (ii) Common standard deviation for difference in cT1, σ = 42.5 ms; (iii) Type I error, α = 0.05 (2-sided significance test) and power = 80% with a 2-sample t-test for mean difference. Eligibility criteria were: Men and women between 18 and 75 years of age with liver biopsies [obtained within 12 months of study] demonstrating NASH with fibrosis stage 3 on the Brunt fibrosis scale [26]. Exclusion criteria included: evidence of other chronic liver diseases (including viral hepatitis B or C, primary biliary cirrhosis (primary sclerosing cholangitis), Wilson’s disease, alpha-1 antitrypsin deficiency, alcoholic hepatitis, hemochromatosis, liver cancer, or history of biliary diversion); HIV infection; significant injury or surgery in previous 8 weeks; weight reduction surgery in past 3 years; significant alcohol consumption (> 20 grams/day for women; > 30 grams/day in men); clinically significant and uncontrolled cardiovascular disease; current infection; history of malignancy in previous 5 years; participation in an investigational drug trial in 30 days before enrolment; any clinically significant psychiatric or medical condition; know allergies to the study drug; pregnancy; contraindications to MRI.

**Fig 1:**
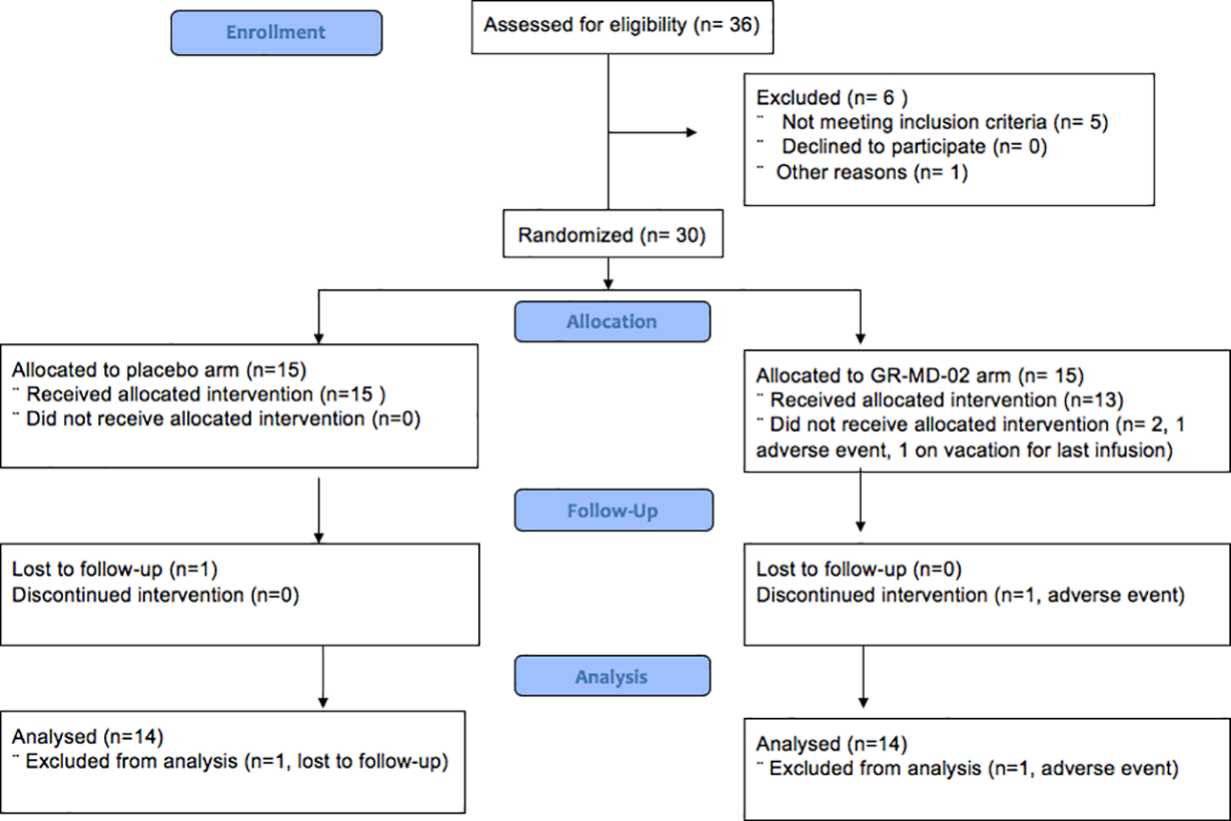
CONSORT flowchart.

All participants gave written informed consent according to the Declaration of Helsinki and as approved by the ethics committee. The study was approved by the Brooke Army Medical Centre (BAMC) Institutional Review Board (IRB) and was registered at clinicaltrials.gov (NCT02421094). Full CONSORT flowchart is included as Fig 1.

### Study design

**Fig 2:**
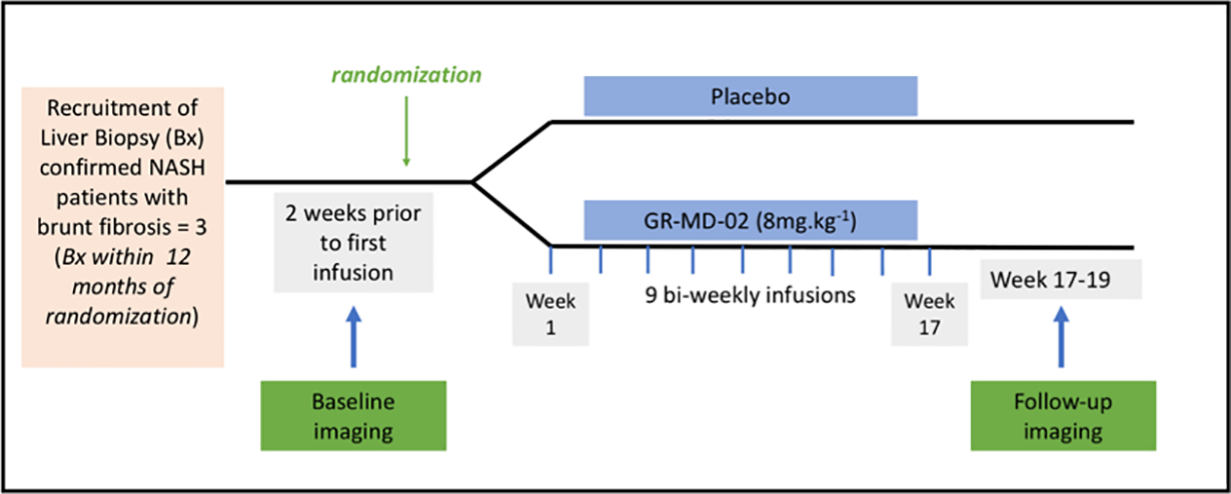
Schematic of the study protocol.

This was a Phase 2, single-centre, parallel group, randomized and controlled double-blind study. Participants with a liver biopsy demonstrating NASH with Brunt stage 3 fibrosis entered screening. The screening visit was up to 6 weeks prior to randomisation and included full medical screening as typically required for clinical trials. Following screening, each subject was randomized 1:1 to receive either placebo or GR-MD-02 at a dose of 8 mg.kg^-1^ lean body mass administered bi-weekly over a 16-week period for a total of 9 intravenous infusions. Drug efficacy was measured using non-invasive imaging modalities at two time-points: Two-weeks prior to infusion visit 1 (Baseline) and within 7–21 days of visit 9 (Follow-up), participants underwent multi-parametric MRI (1.5T, Siemens Avanto, Siemens Healthcare, Germany) with the LiverMultiScan™ (LMS) protocol (Perspectum Diagnostics Ltd, UK) from which cT1 maps, T2* maps and PDFF were calculated. Participants also underwent magnetic MRE (1.5T, Siemens Avanto, Siemens Healthcare, Germany) and LSM (Echosens, France) imaging to assess liver stiffness. The study protocol is represented schematically in Fig 2. Participants fasted for at least 6 hours (water included) prior to the MRI acquisition and the resulting LMS data were processed centrally by Perspectum Diagnostics. MRE data were evaluated by a local radiologist with experience in processing these data. LSM was performed after a 3 hour fast according to the manufacturer’s instructions and evaluated centrally by Echosens. Reviewers of all imaging and liver biopsy data were blinded to study arm allocation.

### Intervention

Lean body mass (LBM) was used for dosing in anticipation that most participants would be obese (BMI ≥ 30) and since GR-MD-02 is distributed primarily in the blood compartment. The dose calculated based on the LBM at the first infusion visit was maintained at each subsequent infusion visit. LBM was estimated from height and weight measurements using well-validated formulae for obese individuals [27]:

•Males:LBM=9270×TBW(totalbodyweightinkg)/(6680+216×BMI)

•Females:LBM=9270×TBW/(8780+244×BMI)

Prior to each infusion subjects underwent: Limited physical examination (including weight, heart, lung and abdominal clinical examination); Vital signs measurements (including heart, and respiratory rate, blood pressure, and body temperature); Hematology, blood chemistry-including alpha-2 macroglobulin, and urine pregnancy test (Visits 1, 5 and 9 only); ECG (Visit 9 only). Treatment compliance was determined by infusion records.

### Outcome measures

The primary objective was to measure the difference in cT1 at follow-up in subjects receiving placebo as compared to those receiving GR-MD-02 (8 mg.kg^-1^ per infusion).

The secondary objectives were to determine the difference in liver stiffness, as determined by MRE and by LSM Score, at follow-up in the same groups, as well as the difference in liver iron and fat (derived from MRI-T2* and MRI-Proton Density Fat Fraction, PDFF), and examine the test-retest repeatability of the three biomarkers of fibrosis.

### Statistical analysis

Statistical analysis was performed using R software version 3.4.3 [28]. Variable distributions were tested for normality (Shapiro-Wilk test) and homogeneity of variance (Levene test). All statistical tests were 2-sided with a 5% level of significance with uncorrected p-values reported.

Difference between the groups in imaging metrics at follow-up were explored using ANCOVA models adjusting for baseline measure (e.g. follow-up cT1 as the outcome variable and baseline cT1 as a covariate). Two types of ANCOVA were performed which used ordinary least squares (OLS) or robust regression. The latter approach was used for variables not normally distributed. The estimated difference (est.difference) between groups at follow-up, as derived from the ANCOVA analysis, is reported for each metric.

To explore the test-retest reliability (repeatability) of cT1, MRE and LSM, the proportion of variation in each measurement due to random error was expressed using percent coefficient of variation (% CoV; standard deviation of the difference between baseline and follow-up divided by mean of all measures). A large CoV indicates large measurement variability.

Missing or uninterpretable data were handled by casewise deletion.

## Results

Thirty subjects were enrolled into the study and equally randomized between GR-MD-02 and placebo arm. The study drug was well tolerated (clinicaltrials.gov (NCT02421094)). Twenty-eight subjects were included in the final analysis with 1 subject from the GR-MD-02 arm stopping after 4 infusions due to an adverse event (erythematous rash) assessed by the study investigator to be unrelated to the infusions, and a subject from the placebo arm being lost to follow up. The placebo and study drug groups were balanced for gender, age, weight and concurrent diagnosis of diabetes. Full demographics can be found in Table 1. Group means for baseline and follow for all variables are in Table 2.

**Table 1:** Participants demographics, mean (SD) unless otherwise stated.

	Placebo (N = 15)	GR-MD-02 (N = 15)
Age (years)	56.7 (0.52)	59.7 (0.51)
Gender (# Female)	7	6
Weight (kg)	102.9 (23.8)	91.5 (20.4)
Height (m)	169.3 (9.7)	166.1 (14.4)
Body Mass Index (BMI)	35.7 (6.9)	32.9 (4.0)
Type 2 diabetes (#)	12	15

**Table 2:** Outcome measures, mean (SD).

	Placebo		GR-MD-02	
	Baseline	Follow-up	Baseline	Follow-up
cT1 (ms)	950.8 (53.9, N = 14)	949.7 (47.3, N = 14)	920.4 (46.3, N = 14)	944.1 (63.9, N = 14)
MRE (kPa)	4.7 (1.2, N = 13)	4.9 (1.4, N = 13)	4.4 (1.7, N = 14)	4.6 (1.8, N = 14)
LSM (kPa)	17.8 (8.9, N = 12)	16.3 (5.9, N = 12)	16.9 (12.1, N = 14)	17.8 (11.2, N = 14)
% PDFF	8.7 (4.8, N = 13)	8.6 (5.9, N = 13)	10.0 (4.6, N = 14)	11.1 (4.4, N = 14)
Liver Iron	1.2 (0.2, N = 14)	1.2 (0.2, N = 14)	1.3 (0.2, N = 14)	1.2 (0.2, N = 14)

cT1 -iron corrected-T1 relaxation time (ms); MRE–liver shear stiffness (kPa) from magnetic resonance elastography; LSM–median liver stiffness (kPa) from shear wave elastography; PDFF–MRI-derived proton density fat fraction (%).

**Fig 3:**
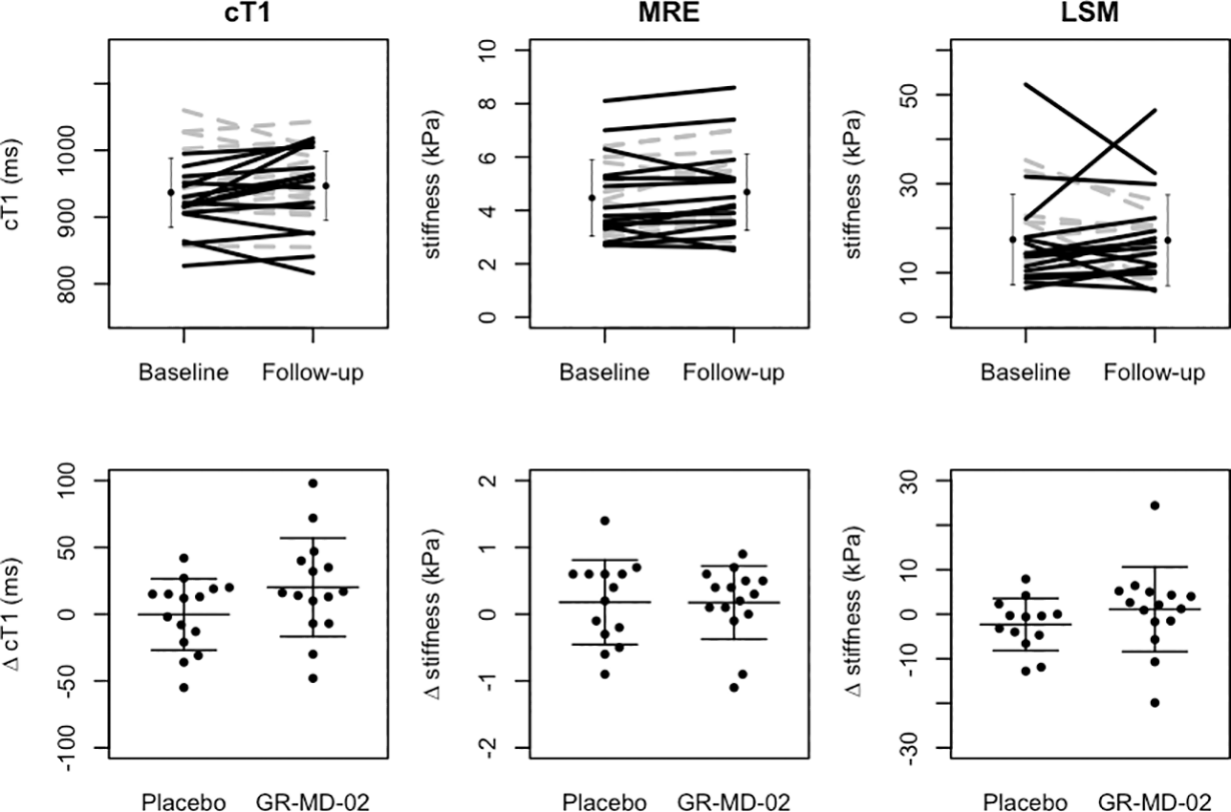
[TOP] Before and after plots for all subjects (placebo represented by the grey dotted lines). Error bars representing the overall SD across both groups. [BOTTOM] Box plots representing difference between treatment groups in change in cT1, MRE and LSM.

The primary outcome measure, cT1, was interpretable for all 28 subjects whom completed the study and cT1 satisfied tests of normality. The ANCOVA test was performed on the group variable (GR-MD-02 v Placebo). The estimated follow-up difference for cT1 between GR-MD-02 and placebo arms was 18.6ms; however, the difference was not statistically significant (t = 1.46, p = 0.16, Fig 3).

In the secondary outcome measure analyses, group differences in MRE and LSM were explored (Fig 3). Neither of these variables was normally distributed at baseline or follow-up. In the MRE dataset, one subject’s data were deemed inadequate and excluded from analysis. In the LSM dataset, data from two subjects were uninterpretable from one of the time points and excluded from analysis.

Analysis of the remaining MRE data (n = 27) showed no significant difference in shear stiffness between groups at follow-up (est. difference 0.07, t = 0.26, p = 0.80); Similarly, there was no significant difference in LSM between study groups (n = 26, est. difference 1.5 kPa, t = 0.49, p = 0.63).

For the additional biomarkers of liver iron and liver fat, t2* images were acquired adequately for liver iron quantification in all 28 cases, however one subject’s PDFF value on follow-up was not acquired correctly, thus only the 27 cases were analysed. PDFF satisfied tests of normality, however iron did not, thus iron was analysed with robust one-way ANCOVA. There was no significant difference at follow-up between the study groups for either liver fat (GR-MD-02-placebo estimate = 1.37%, t = 0.32, p = 0.75), or liver iron (est. difference 0.08 kPa; t = 1.44, p = 0.16).

To further investigate the relationship between the three main biomarkers, a supplemental ANCOVA analysis was conducted for each one, adjusting for the influence of the other biomarkers in turn. Repeat analysis of the group difference in cT1, when adjusting for liver stiffness (MRE or LSM) revealed a significant group effect on cT1 (MRE: GR-MD-02-placebo estimate = 26 ms, t = 2.40, p = 0.025; LSM: GR-MD-02-placebo estimate = 27 ms, t = 2.6, p = 0.016). In contrast, analysis of the group different in liver stiffness (MRE or LSM) adjusting for cT1 as covariate did not result in a significant group effect (MRE: GR-MD-02-placebo estimate = 0.11 kPa, t = 0.41, p = 0.51; LSM: GR-MD-02-placebo estimate = 0.9 kPa, t = 0.41, p = 0.69).

Correlation analysis was performed to further explore the relationships between biomarkers. Results showed the two elastography measures, MRE and LSM, correlated strongly (r = 0.86, p < 0.001) however, cT1 showed weak and non-significant correlations with both liver stiffness metrics (MRE, r = 0.25, p = 0.18; LSM, r = 0.31, p = 0.11). We observed a moderate and significant negative correlation between % Liver Fat (PDFF) and MRE (r = -0.45, p = 0.013), and a moderate, but non-significant, correlation between Fat and LSM (r = -0.36, p = 0.06). There was a strong, positive significant correlation between % liver fat and with liver iron content (r = 0.62, p = 0.0003) and a weak significant correlation between % liver fat and cT1 (r = 0.37, p = 0.046).

The coefficient of variation (% CoV) for each of the three main biomarkers is displayed in Table 3. The analysis was initially done on the results from the placebo group only, given no change expected in any of the biomarkers. Results revealed test-retest for cT1, MRE and LSM of 2.3%, 13.5% and 32% respectively. Since no superior efficacy over placebo was shown for GR-MD-02, data from both treatment arms were pooled and the analysis repeated. Overall % CoV for test-retest for cT1, MRE and LSM were 3.1%, 11%, 40% and respectively.

**Table 3:** Coefficient of variation of the three biomarkers of fibrosis.

	cT1	MRE	LSM
Placebo group	2.3%	13.5%	32%
Combined	3.1%	11%	40%

cT1 -iron corrected-T1 relaxation time (ms); MRE–liver shear stiffness (kPa) from magnetic resonance elastography; LSM–median liver stiffness (kPa) from shear wave elastography; CoV is the ratio of the standard deviation to the mean, lower CoV represents lower variation between repeated measures.

## Discussion

In this study, we showed that there was no significant difference in the primary outcome measure, cT1, between the placebo and GR-MD-02 groups following a 4-month intervention period. Similarly, there was no significant difference in liver stiffness (measured by MRE or shear wave elastography) between the GR-MD-02 and placebo arms, or in either percent liver fat or liver iron content between the groups at follow-up.

There was a very strong correlation between the two elastrographic metrics but cT1 was only weakly correlated with either. cT1 was also only weakly correlated with PDFF, similar to LSM. These findings, in light of previously published utility of these other biomarkers [9,29–31], suggest together cT1 with elastography and PDFF may provide a more comprehensive and complementary characterisation of liver disease. This complementarity was further supported by an ANCOVA analysis which indicated that cT1 provides additional information on tissue characteristics that is not captured by elastography. Given the heterogeneity in both clinical presentation and histological severity, coupled with a desire for reduced reliance on liver biopsy, and the rising worldwide prevalence of NAFLD, non-invasive methods that can quantitatively characterize the broad spectrum of hepatic abnormalities are becoming increasing more popular [32]. Over the past decade there has been a surge in research into serum [33] and radiological [34] biomarkers of liver disease, and it is increasingly recognised that combining information from individual biomarkers to comprehensively characterize disease can greatly improve screening, diagnostic and responsiveness accuracy [31,33,35,36].

Examination of the repeatability of the biomarkers showed cT1 to have far greater test-retest reliability than MRE or LSM, suggesting cT1 is a suitable measure for longitudinal monitoring of change in patients with NASH with advanced fibrosis. By comparison, direct measures of mechanical liver stiffness using MRE or LSM methods each showed great variability with contributions from random error. cT1 measures were interpretable in all cases, whereas elastography data from three of twenty-eight subjects (one with MRE and two with LSM) were deemed inadequate and excluded from analysis. These exclusions may reflect high levels of obesity within the study population (mean BMI = 35 kg.m^2^, range 25.5–52.7), a commonly reported contraindication for successful liver stiffness measurements with LSM [23].

Results from both animal studies [6,7] and from a phase 1 human trial [8] suggested a possible efficacy of GR-MD-02 in reducing fibrosis in NASH. However, the promise of drug from the human phase 1 trial came from a dose dependent reduction in FibroTest® (a biomarker test that uses the results of six blood serum tests to generate a score that has been showed to correlate with the degree of liver damage in people with a variety of liver diseases [9], believed to be as a result of a reduction in alpha- 2 macroglobulin levels. Actual mechanical, liver specific test of fibrosis only showed a trend for a reduction in fibrotic tissue following treatment with GR-MD-02. The results of the 12-week course of 8 mg.kg^-1^ GR-MD-02 reported here, may be interpreted as indicating the efficacy for this drug as a therapeutic for liver fibrosis potentially lacks sensitivity to high levels of fibrosis, or was insufficiently dosed in the current trial. In a larger subsequent study of the efficacy of GR-MD-02 in 162 patients with NASH with cirrhosis and portal hypertension, with liver biopsy and hepatic venous pressure gradient (HPVG) as endpoints [37], a pre-specified group of patients with mild portal hypertension showed a significant decrease in HVPG following a year of treatment with 2 mg.kg^-1^ GR-MD-02. Furthermore GR-MD-02 dosed at both 2 and 8 mg.kg^-1^ resulted in improved hepatocyte ballooning in the total population, correlating with an improvement in HVPG. The link between the concomitant decrease in portal pressure and improvement in ballooning is hypothesised to reflect a decrease in liver cell death.

It should be noted that this study was limited by the small number (n = 30) of subjects with advanced liver disease that may not show clinically significant changes in necro-inflammatory activity over a short duration (16 weeks). Additionally, because liver biopsies were not included in this study, histological assessment of any change in NASH-related pathologies was not possible. Furthermore, as the use of cT1 as a biomarker of pharmacodynamic efficacy is still in its infancy, largely due to the limited number of successful NASH trials, we look forward to better understanding its sensitivity to change.

## Conclusions

In conclusion, a 16-week course of biweekly infusions of 8 mg.kg^-1^ GR-MD-02 had no statistically-significant effect on non-invasive biomarkers of liver inflammation or fibrosis over a 4-month intervention period. The primary outcome measure, cT1, had excellent reproducibility (CoV = 3.1%). This low variability of cT1 is important for detecting even small changes in fibro-inflammatory activity in the setting of clinical trials in NASH. Moreover, cT1 was reliably assessed in all subjects and has utility in assessing longitudinally future short-term and long-term study treatments in NASH.

## Supporting information

S1 Checklist CONSORT checklist. (PDF)

S1 Dataset Full study dataset. (CSV)

S1 Protocol GT-028 NASH-FX full protocol. (PDF)
