# Supplementary material for: Utility and variability of three non-invasive liver fibrosis imaging modalities to evaluate efficacy of GR-MD-02 in subjects with NASH and bridging fibrosis during a phase-2 randomized clinical trial
Source: PLoS One. 2018 Sep 7;13(9):e0203054. doi: 10.1371/journal.pone.0203054 (PMC6128474; doi:10.1371/journal.pone.0203054)
Supplement: S1 Protocol — (PDF) [file pone.0203054.s003.pdf]

CLINICAL STUDY PROTOCOL
IND Number 115459

A Randomized, Controlled, Double-blind, Parallel Group, Single Center Phase 2 Clinical Trial to Evaluate Multiple Non-Invasive Liver Fibrosis Imaging Methods in the Assessment of the Efficacy of GR-MD-02 for the Treatment of Liver Fibrosis in Patients with NASH with Advanced Fibrosis

Study GT-028

The NASH-FX Study

**Version of Protocol:**	GT-028, Version 4.0
**Date of Protocol:**	January 27, 2016
**Previous Date(s) and Versions(s):**	Original: March 31, 2015, Version 1.0<br>Amendment 1: July 24, 2015<br>Amendment 2: September 14, 2015

Table of Contents

Table of Contents 2
Protocol Synopsis 6
List of Abbreviations 12
1 Introduction 14
2 Study Objectives 16
2.1 Primary Objective(s) 16
2.2 Secondary Objective(s) 16
2.3 Exploratory Objectives 16
2.4 Safety objectives 16
3 Investigational Plan 17
3.1 Study Design 17
3.1.1 Rationale of Study Design 19
4 Subject Selection and Withdrawal Criteria 20
4.1 Selection of Study Population 20
4.1.1 Inclusion Criteria 20
4.1.2 Exclusion Criteria 21
4.2 Withdrawal of Subjects From the Study 23
4.2.1 Reasons for Withdrawal/Discontinuation 23
4.2.2 Handling of Withdrawals 24
4.2.3 Replacements 24
5 Study Treatments 25
5.1 Method of Assigning Subjects to Treatment Groups 25
5.2 Identity of Investigational Product 26
5.3 Management of Clinical Supplies 27
5.3.1 Study Drug Packaging and Storage 27
5.3.2 Test Article Accountability 27
5.3.3 Other Supplies 27

5.4 Overdose Management ... 28
5.4.1 Treatment of Overdose ... 28
5.4.2 Medication Errors ... 28
5.4.3 Treatment of Medication Errors ... 29
5.5 Misuse for Illegal Purposes ... 29
5.6 Blinding ... 29
5.6.1 Breaking the Blind ... 29
5.7 Treatment Compliance ... 30
5.8 Prior and Concomitant Therapy ... 30
5.9 Prohibited Medications or Therapies ... 30
6 Study Assessments and Procedures ... 31
6.1 Study Visits ... 31
6.1.1 Screening Visit (Week -6 to Day 0) ... 31
6.1.2 Treatment Phase ... 32
6.1.2.1 Randomization ... 32
6.1.2.2 Two Weeks Prior to Infusion Visit 1 ... 32
6.1.2.3 Infusion Visit 1 (Week 1), Infusion Visit 2 (Week 3 ± 3 days), Infusion Visit 3 (Week 5 ± 3 days), Infusion Visit 4 (Week 7 ± 3 days), Infusion Visit 5 (Week 9 ± 3 days), Infusion Visit 6 (Week 11 ± 3 days), Infusion Visit 7 (Week 13 ± 3 days), Infusion Visit 8 (Week 15 ± 3 days), Infusion Visit 9 (Week 17 ± 3 days) ... 33
6.1.3 Follow-up study evaluations (7-21 Days after Final Dose) ... 33
6.1.4 Follow-up/Early Termination ... 34
6.2 Efficacy Assessments ... 34
6.2.1 LiverMultiScan ... 34
6.2.2 MR Elastography ... 34
6.2.3 FibroScan ... 35
6.3 Safety Assessments ... 35
6.3.1 Vital Signs ... 35
6.3.2 Physical Examination ... 35
6.3.3 Electrocardiogram ... 36
6.3.4 Adverse Events ... 36
6.3.4.1 Definitions of Adverse Events ... 36
6.3.4.2 Eliciting and Documenting Adverse Events ... 36

6.3.4.3 Reporting Adverse Events ... 37
6.3.4.4 Assessment of Severity ... 38
6.3.4.5 Assessment of Causality ... 38
6.3.4.6 Exceptions ... 39
6.3.4.7 Follow-Up of Subjects Reporting Adverse Events ... 39
6.3.5 Pregnancy ... 39
6.3.6 Laboratory Analyses ... 40
6.3.7 Hematology ... 41
6.3.8 Blood Chemistry ... 41
6.3.9 Urinalysis ... 41
6.4 Sample Collections ... 42
7 Statistical and Analytical Plan ... 43
7.1 Primary Efficacy Endpoint ... 43
7.2 Secondary Efficacy Endpoints ... 43
7.3 Exploratory Endpoints ... 43
7.4 Safety Endpoints ... 43
7.5 Sample Size Calculations ... 43
7.6 Analysis Sets ... 44
7.7 Description of Subgroups to be Analyzed ... 45
7.8 Statistical Analysis Methodology ... 45
7.8.1 Analysis of Primary Efficacy Endpoint ... 45
7.8.2 Analysis of Key Secondary Efficacy Endpoints ... 46
7.8.3 Analysis of the Exploratory Efficacy Endpoint ... 46
7.8.4 Safety Analyses ... 46
7.8.5 Other Analyses ... 47
7.8.6 Interim Analyses ... 48
7.9 Data Quality Assurance ... 48
7.9.1 Data Management ... 48
8 Ethics ... 50
8.1 Independent Ethics Committee or Institutional Review Board ... 50
8.2 Ethical Conduct of the Study ... 50
8.3 Subject Information and Consent ... 50

9 Investigator's Obligations 52
9.1 Confidentiality 52
9.2 Financial Disclosure and Obligations 52
9.3 Investigator Documentation 53
9.4 Study Conduct 53
9.5 Adherence to Protocol 53
9.6 Adverse Events and Study Report Requirements 54
9.7 Investigator's Final Report 54
9.8 Records Retention 54
9.9 Publications 54
10 Study Management 55
10.1 Monitoring 55
10.1.1 Monitoring of the Study 55
10.1.2 Inspection of Records 55
10.2 Management of Protocol Amendments and Deviations 55
10.2.1 Modification of the Protocol 55
10.2.2 Protocol Deviations 56
10.3 Study Termination 56
10.4 Final Report 57
11 Schedule of Events 58
12 References 61
13 Protocol Amendments 63
13.1 Amendment 1 63
13.2 Amendment 2 65
13.3 Amendment 3 66

Protocol Synopsis

**Protocol Number:**	GT-028
**Title:**	A Randomized, Controlled, Double-blind, Parallel Group, Single Center Phase 2 Clinical Trial to Evaluate Multiple Non-Invasive Liver Fibrosis Imaging Methods in the Assessment of the Efficacy of GR-MD-02 for the Treatment of Liver Fibrosis in Patients with NASH with Advanced Fibrosis. (The NASH-FX Study)
**Sponsor:**	Galectin Therapeutics Inc.<br>4960 Peachtree Industrial Blvd, Suite 240<br>Norcross, GA 30071
**Study Phase:**	Phase 2
**Study Sites:**	Single site in the U.S.
**Indication:**	Non-Alcoholic Steatohepatitis (NASH) with advanced fibrosis
**Rationale:**	NASH is a chronic inflammatory disease of the liver characterized by progressive fibrosis leading eventually to cirrhosis in a subset of patients, the complications of which can lead to death or liver transplantation. As there are currently no medical therapies approved for NASH or for liver fibrosis, this program has received Fast Track designation from the FDA. Galectin-3, a galactose binding protein, has been shown to be critical in the pathophysiology of NASH and liver fibrosis. GR-MD-02, a complex carbohydrate drug that binds to galectin-3, has shown robust efficacy in pre-clinical models of NASH and liver fibrosis, as well as disease marker effects in a Phase 1 human trial. Therefore, the overall objective of this clinical trial is to establish the efficacy of GR-MD-02 as compared to placebo in the reduction liver stiffness in NASH subjects with advanced fibrosis.

Objectives:

The primary objective is to determine the difference between placebo and GR-MD-02 treatment in the baseline adjusted mean change in liver fibrosis as measured by corrected T1 (cT1) mapping as determined from LiverMultiScan (LMS), a multi-parametric MRI protocol.

Secondary objectives include evaluating differences between subjects treated with GR-MD-02 versus placebo in:

- The baseline-adjusted change in liver stiffness as measured by MR-elastography
- The baseline-adjusted change in liver stiffness as measured by FibroScan® scores.

An exploratory objective will be to evaluate the correlation of the three diagnostic modalities of LiverMultiScan, MR-Elastography, and FibroScan®. Additionally, serum will be analyzed for alpha-2 macroglobulin.

Safety objectives include determining the:

- Incidence of adverse events during study treatment
- Emergent physical examination abnormalities
- Emergent laboratory parameter abnormalities

Subject Population:

Subjects will be entered into the study and randomized if they have a liver biopsy confirmed diagnosis of NASH with advanced fibrosis as defined by Brunt stage 3 fibrosis within 12 months of randomization.

Additional inclusion criteria (detailed in protocol) will include age ≥ 18 years and ≤ 75 years old at the time of screening, ability to provide written informed consent, and reproductive status.

Exclusion criteria (detailed in protocol) will include in excess of defined level of alcohol consumption, weight reduction surgery within 3 years, history of hepatic decompensation, evidence of other forms of liver disease, laboratory values indicative of advanced liver disease, various concomitant medical illnesses (HIV infection, recent major surgery, uncontrolled heart disease, concurrent infection or fever of unknown origin, illicit drug use, significant malignant disease), participation in an investigational new drug (IND) trial in the 30 days before randomization, clinically significant medical or psychiatric condition considered a high risk for participation in an investigational study, failure to give informed consent, and subjects with known allergies to the study drug or any of its excipients.

**Study Design:**	Study GT-028 is a phase 2 is a randomized, controlled, double blind, parallel group and single US center study of subjects with NASH with advanced fibrosis (Brunt stage 3). All subjects are required to have signed Institutional Review Board (IRB) or Ethics Committee (EC)-approved informed consent prior to undergoing any study specific procedures<br><br>Eligible subjects will be randomized (1:1) to receive one of two treatment assignments including GR-MD-02 in dose of 8 mg/kg lean body mass or placebo administered every other week over a 16 week period for a total of 9 infusions. The primary endpoint will be evaluation of the baseline adjusted mean change in liver fibrosis as measured by c T1 mapping as determined from LMS, at 17-19 weeks following the first infusion. Change in from baseline will be compared between treatments groups using analysis of covariance adjusted for baseline cT1 at screening.<br><br>Key secondary endpoints will include two other non-invasive assessments of liver fibrosis for comparison with LMS, including MR-elastography (MRE) and FibroScan (FS). LMS, MRE and FS will be performed within two weeks of the first infusion and within 7-21 days after the final 9th dose of study drug. Additionally, serum will be analyzed for alpha-2 macroglobulin.<br><br>All subjects are to attend a final study visit 30 days after the last dose (or at the time of early discontinuation) to evaluate safety.<br><br>Following study completion, subjects will be offered enrollment into a subsequent separate study, an open-label extension study (OLES), if there is adequate tolerability, no safety issues, no signs of clinical progression that would require discontinuation, and evidence of improvement on the primary endpoint in the study.
**Estimated Study Duration:**	Subjects will remain on study therapy for a total of sixteen (16) weeks unless intolerable side effects develop, or the subject is withdrawn from study participation. Subjects may be discontinued at the discretion of the investigator.

**Efficacy Assessments:**	The primary efficacy assessment will be the determination of the baseline-adjusted change in cT1 as assessed by LMS following 16 weeks of therapy with GR-MD-02 as compared to placebo. Key secondary endpoints will include two other non-invasive assessments of liver fibrosis for comparison with LMS including the MRE score and FS. Additionally, serum will be analyzed for alpha-2 macroglobulin.
**Pharmacokinetic Assessments:**	None
**Safety Assessments:**	Safety assessments will include, incidence of adverse events during study treatment, emergent physical examination abnormalities, emergent vital sign and ECG abnormalities, and laboratory parameter abnormalities.<br>All subjects receiving any part of at least one injection of study treatment will be evaluated for safety. The safety analyses will include evaluation of the incidence of treatment-emergent adverse events, Grade 3 or greater adverse events, serious adverse events and adverse events leading to discontinuation of study treatment. Laboratory and vital signs assessments will be evaluated over time on study using descriptive statistics. Shift analyses of relevant clinical laboratory parameters will be produced showing shifts across low, normal, and high categories.
**Study Drug, Dosage, and Route of Administration:**	Subject arms will be randomized to receive or 8 mg/kg lean body weight (up to a maximum of 800 mg total) of GR-MD-02 or placebo, in a 1:1 ratio. The study drug will be diluted in 100 ml of normal saline and infused intravenously via a peripheral vein over 60 min. The study drug will be administered every other week for 16 weeks for a total of 9 doses. No dose modification for GR-MD-02 is allowed. Subjects in the placebo arm will receive placebo diluted in normal saline administered in the same fashion. Drug and placebo will be blinded by using intravenous bag covers and colored tubing.
**Sample Size:**	A total of 30 subjects will be randomized into two parallel treatment arms in a 1:1 ratio. Subjects who are screened but not randomized may be replaced by additional new subjects.

Statistical Methods:

All statistical tests will be two-sided and will be at the 5% level of significance. Unless otherwise specified, continuous variables will be summarized by randomized treatment group with the number of non-missing observations, mean, standard deviation, median, 25th and 75th percentile displayed. Categorical data will be summarized by randomized treatment group as counts and percentages.

Analysis of the primary and secondary efficacy endpoints will be based on the ITT analysis set. Supportive sensitivity analyses of these endpoints will be performed using the Per Protocol analysis set, treated subjects with no major protocol deviations.

The primary efficacy endpoint analysis is the baseline-adjusted change in cT1 score at following treatment for 16 weeks with placebo as compared to subjects treated with GR-MD-02 (8 mg/kg/week). Change in cT1 as determined by LMS from baseline will be compared between treatments groups using analysis of covariance adjusted for baseline cT1 score at screening. Secondary endpoints will be analyzed in a similar fashion.

All subjects receiving any part of at least one injection of study treatment will be evaluated for safety, including evaluation of the incidence of treatment-emergent adverse events, Grade 3 or greater adverse events, serious adverse events and adverse events leading to discontinuation of study treatment. Laboratory and vital signs assessments will be evaluated over time on study using descriptive statistics. Shift analyses of relevant clinical laboratory parameters will be produced showing shifts across low, normal, and high categories.

A total sample size of 30 patients will be enrolled in the two groups based on the following assumptions: 1) True mean change in cT1 from baseline of 0 in the placebo group; 2) true mean change in cT1 of 53 ms (milliseconds) from baseline in the GR-MD-02 dose group; 3) common standard deviation of 50 ms for difference in cT1; 4) null hypothesis, H0: $\theta = \Delta G - \Delta p = 0$; 5) type I error, $\alpha = 0.05$ (two-sided significance test); 6) power = 80%; 7) statistical test = two-sample t-test for mean difference 8) randomization ratio = 1:1

Date of Protocol: September 14, 2015

List of Abbreviations

Abbreviation	Definition
AE	adverse event
ALT	alanine aminotransferase
ANCOVA	analysis of co-variance
AP	alkaline phosphatase
AST	aspartate aminotransferase
BMI	body mass index
BUN	blood urea nitrogen
CFR	Code of Federal Regulations
CI	confidence interval
CRF	case report form
cT1	corrected T1
CV	curriculum vitae
ECG	Electrocardiogram
eCRF	electronic case report form
FAS	full-analysis set
FDA	Food and Drug Administration
FS	FibroScan
GCP	Good Clinical Practice
GGT	gamma-glutamyltransferase
GI	Gastrointestinal
HBsAg	hepatitis B surface antigen
Hct	Hematocrit
Hgb	Hemoglobin
HIV	human immunodeficiency virus
ICF	informed consent form
ICH	International Conference on Harmonisation
IEC	independent ethics committee
IND	investigational new drug
IRB	institutional review board
ITT	intent to treat
IVRS	interactive voice response system

Abbreviation	Definition
LDH	lactate dehydrogenase
LLN	lower limit of normal
LMS	LiverMultiScan
LOCF	last observation carried forward
MRE	magnetic resonance elastography
MRI	magnetic resonance imaging
MedDRA	Medical Dictionary for Regulatory Activities
ms	milliseconds
NDA	new drug application
NSAID	nonsteroidal anti-inflammatory drug
OTC	over-the-counter
PD	pharmacodynamic
PK	pharmacokinetic
PP	per protocol
PPS	per-protocol set
RBC	red blood cell
SAE	serious adverse event
TEAE	treatment-emergent adverse event
T_{max}	time to reach the observed maximum (peak) concentration
ULN	upper limit of normal
WBC	white blood cell

1 Introduction

Nonalcoholic fatty liver disease (NAFLD), or fatty liver, and nonalcoholic steatohepatitis (NASH) are common liver disorders in the United States (US). The major feature in NAFLD is fat accumulation in hepatocytes with minimal inflammation. It is estimated that the worldwide prevalence of NAFLD ranges from 6.3% to 33% with a median of 20% in the general population (1), but a recent study in asymptomatic middle-aged adults in the US suggests that it may be as high as 46% (2). A subset of individuals with NAFLD are found to have NASH, which is characterized by excessive fat accumulation in hepatocytes (steatosis) with the addition of inflammatory cell infiltrates, evidence of damage to hepatocytes (ballooning degeneration), and the deposition of fibrous tissue. It is estimated that between 3 to 5% of Americans are affected by NASH (1), with as many as 12.2 % in asymptomatic adults (2). While the prevalence of cirrhosis in NASH is not clearly defined, as many as one third of NASH subjects progress to advanced fibrosis (3). The only therapy available to these advanced subjects is liver transplantation. Currently the percentage of liver transplantations performed in the US for NASH is between 10 and 15%, but the numbers are increasing and it has been suggested that it may become the leading cause for liver transplantation over the next 20 years (4).

The galectin-3 protein has recently been implicated in the pathogenesis of NASH. Galectins are a family of proteins, containing 15 members (11 identified in humans), which have the property of binding avidly to galactose containing oligosaccharides associated with glycoproteins (5). GR-MD-02 (galactoarabino-rhamnogalaturonan) represents a new type of agent for the therapy of subjects with NASH and advanced fibrosis. GR-MD-02 is a complex carbohydrate molecule derived from a natural plant compound which contains oligosaccharide chains containing galactose residues and binds to galectin-3, and to a lesser extent to galectin-1. Cellular experiments demonstrate that GR-MD-02 is not toxic to cells, but is capable of inhibiting the expression of inflammatory cytokines in a monocyte/macrophage model of inflammation and reduces cell surface galectin-3 from fibrogenic liver stellate cells.

GR-MD-02 has been tested in 2 models of liver fibrosis, a mouse model that reliably produced a pathological picture of NASH with a fibrosis and a toxin-induced model of liver fibrosis in rats. Multiple studies were completed in the mouse NASH model that showed that GR-MD-02 consistently reduced the activity of NASH, reduced or eliminated fibrosis as

measured by liver collagen, and reduced the expression of galectin-3 in liver macrophages (6). Much more robust fibrosis and cirrhosis were induced in rats treated with thioacetamide in which animals developed fibrosis that replaced 25% of the liver with collagen and had all the pathological characteristics of cirrhosis. Treatment of cirrhotic rats with 4 weekly doses GR-MD-02 resulted in reduction of collagen to below 10%, reversal of cirrhosis, and reduced portal hypertension (7).

The results from a Phase 1 study indicated GR-MD-02 was safe and well tolerated at single and multiple doses of 2, 4, and 8 mg/kg. Pharmacokinetics revealed drug exposure in humans at the 8 mg/kg dose that was equivalent to the upper range of the targeted therapeutic dose determined from effective doses in NASH animal models, thus providing support for the proposed Phase 2 dosing regimen. There was evidence of an effect on a relevant disease marker, with a dose dependent reduction in FibroTest® (FibroSure®) scores due to a reduction in alpha-2 macroglobulin levels. Additionally, there was a signal of reduced liver stiffness as assessed by FibroScan® in subjects treated with 8 mg/kg GR-MD-02.

2 Study Objectives

2.1 Primary Objective(s)

The primary objective is to determine the difference between four-month treatment with placebo and GR-MD-02 in the baseline adjusted mean change in liver fibrosis as measured by corrected T1 (cT1) mapping as determined from LiverMultiScan (LMS), a multi-parametric MRI protocol.

2.2 Secondary Objective(s)

The secondary objectives of this study is to compare differences in relevant assessments of fibrosis and cirrhosis between GR-MD-02 treated and placebo treated subjects. Secondary objectives will be evaluated using hierarchical serial gatekeeping approach if the primary endpoint reaches statistical significance (described in statistical methods). If the primary endpoint or one of the secondary endpoints does not reach statistical significance, the analysis of ensuing endpoints will be exploratory only. The secondary objects include:

- To determine the baseline-adjusted change in MRE (as measured in kPa) after 4 months of therapy
- To determine the baseline-adjusted change in FS Score after 4 months of therapy

2.3 Exploratory Objectives

An exploratory objective will be to evaluate the correlation of the three diagnostic modalities of LiverMultiScan, MR-Elastography, and FibroScan®. Additionally, serum will be analyzed for alpha-2 macroglobulin.

2.4 Safety objectives

- Incidence of adverse events during study treatment
- Emergent physical examination abnormalities
- Emergent laboratory parameter abnormalities

3 Investigational Plan

3.1 Study Design

Study GT-028 is a phase 2, single-center, parallel group, randomized and controlled double blind study of subjects with NASH with advanced fibrosis (Brunt stage 3). All subjects are required to have signed Institutional Review Board (IRB) or Ethics Committee (EC)-approved informed consent prior to undergoing any study specific procedures.

Below is a graphical representation of the study design. This study will enroll subjects with a liver biopsy showing NASH with Brunt stage 3 fibrosis. Subjects will be randomized (1:1) to receive either placebo or GR-MD-02 in a dose of 8 mg/kg lean body mass administered every other week over a 16 week period for a total of 9 infusions. The primary endpoint will be evaluation of the baseline-adjusted change in cT1 as assessed by LMS at 17-19 weeks following the first infusion.

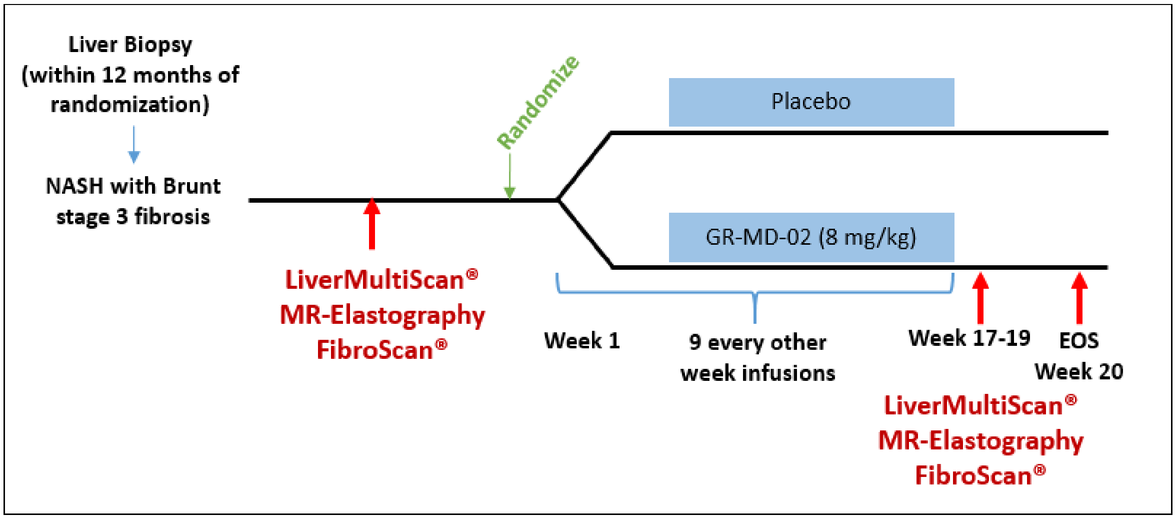

<u>Screening:</u>

Subjects who have had a liver biopsy performed within the previous 12 months which demonstrated NASH with Brunt stage 3 fibrosis, and meet the other inclusion and exclusion criteria, will have LMS, MRE (done at the same time as the LMS), and FS. At the time of screening, subjects will have the following performed:

- Medical, surgical and medication history

- Vital signs (cardiac and respiratory rate, blood pressure and body temperature), complete physical examination, including height and weight
- Electrocardiogram (ECG)
- Clinical laboratory evaluations (hematology, chemistry, coagulation profile, viral hepatitis B and C serology, HIV serology, urinalysis, and pregnancy test [for women of childbearing potential].

Randomization

Subjects will be entered into the study and randomized if they have both a liver biopsy diagnosis of NASH with Brunt stage 3 fibrosis within 12 months of study start and meet the other inclusion and exclusion criteria and have had baseline LMS, MRE, and FS. Allocation of subjects will be to placebo or GR-MD-02 in a dose of 8 mg/kg lean body mass in a ratio of 1:1 and subjects treated every other week for 16 weeks for a total of 9 infusions. The primary efficacy endpoint analysis is the baseline-adjusted change in cT1 as assessed by LMS as measured at 17-19 weeks after the first infusion. Change in cT1 from baseline will be compared between treatments groups using analysis of covariance adjusted for baseline liver stiffness at screening (see Statistical Methods).

Study procedures

During the treatment phase of the trial, all subjects randomized to study therapy will attend study visits according to a schedule of visits for study administration and monitoring. Study assessments performed prior to each drug infusion will include vital signs, limited physical examination, and assessment of adverse events and concomitant medications. Additionally, clinical laboratory tests, urinalysis, and a urine pregnancy test will be performed at the first, fifth, and ninth infusion visits.

Within two weeks of the first infusion and within 1 to 3 weeks after the last infusion subjects will undergo LMS, MRE, and FS.

The Principal Investigator will make all decisions regarding withdrawal of subjects from the trial due to clinical events.

All subjects are to attend a final study visit 30 days after the last dose (or at the time of early discontinuation) to evaluate safety. During this visit, a final physical exam will be performed, vital signs and weight will be measured, and adverse events, if applicable, evaluated.

Open-Label Extension Study

Following study completion, subjects will be offered enrollment into a subsequent separate study, an open-label extension study (OLES), if there is adequate tolerability, no safety issues, no signs of clinical progression that would require discontinuation, and evidence of improvement on the primary endpoint in the study.

3.1.1 Rationale of Study Design

NASH is a chronic inflammatory disease of the liver characterized by progressive fibrosis leading eventually to cirrhosis in a subset of patients, the complications of which can lead to death or liver transplantation. As there are currently no medical therapies approved for NASH or for liver fibrosis, this program has received Fast Track designation from the FDA. Galectin-3, a galactose binding protein, has been shown to be critical in the pathophysiology of NASH and liver fibrosis. GR-MD-02, a complex carbohydrate drug that binds to galectin-3, has shown robust efficacy in pre-clinical models of NASH and liver fibrosis. Therefore, the overall objective of this clinical trial is to establish the safety and efficacy of GR-MD-02 as compared to placebo in the reduction of liver fibrosis as determined by LMS in patients with advanced fibrosis due to NASH.

4 Subject Selection and Withdrawal Criteria

4.1 Selection of Study Population

Approximately 30 subjects will be enrolled at Brooke Army Medical Center at Fort Sam Houston, TX in the United States. Subjects will be assigned to study treatment only if they meet all of the inclusion criteria and none of the exclusion criteria.

Deviations from the inclusion and exclusion criteria are not allowed because they can potentially jeopardize the scientific integrity of the study, regulatory acceptability, or subject safety. Therefore, adherence to the criteria as specified in the protocol is essential.

4.1.1 Inclusion Criteria

Each subject must meet all of the following criteria to be enrolled in this study:

1. Subjects must have liver biopsy demonstrating NASH with Brunt Stage 3 fibrosis within 12 months of randomization. The subject is ≥ 18 years of age and ≤ 75 years old at the time of screening.
2. The subject is willing and able to provide written informed consent.
3. The subject is not pregnant and must have a negative pregnancy test prior to start of the study. Post-menopausal women must have been amenorrheic for at least 12 months to be considered of non-child-bearing potential.
4. Fertile men and women participating in heterosexual relations must agree to use effective means of contraception (i.e., 2 effective methods of contraception, 1 of which must be a physical barrier method; effective forms of contraception include: condom, hormonal methods [birth control pills, injections or implants], diaphragm, cervical cap, or intrauterine device) throughout their participation in this study and for 90 days after discontinuation of study medication. Surgically sterile males and females are not required to use contraception provided they have been considered surgically sterile for at least 6 months. Surgical sterility includes history of vasectomy, hysterectomy, bilateral salpingo-oophorectomy, or bilateral tubal ligation. Post-menopausal women who have been amenorrheic for at least 2 years at the time of screening will be considered sterile.

5. Lactating females must agree to discontinue nursing before the start of study treatment and refrain from nursing until 90 days after discontinuation of study medication.
6. Male subjects must refrain from sperm donation throughout the study period and for a period of 90 days following the last dose of study drug.

4.1.2 Exclusion Criteria

Subjects meeting any of the following criteria will be excluded from the study:

1. A history of hepatic decompensation including any episode of variceal bleeding, clinically detectable ascites, or overt hepatic encephalopathy.
2. Status post TIPS (Transjugular Intrahepatic Porto-systemic Shunt) procedure.
3. Evidence of other forms of chronic liver disease including viral hepatitis B or C, primary biliary cirrhosis, primary sclerosing cholangitis, Wilson's disease, alpha-1 antitrypsin deficiency, alcoholic hepatitis, hemochromatosis, liver cancer, or history of biliary diversion.
4. Any of the following laboratory values:
    a. Serum alanine aminotransferase (ALT) and aspartate aminotransferase levels > 10X upper limits of normal
    b. Serum creatinine $\geq$ 2.0 mg/dL
    c. Platelet count < 60,000/mm3
    d. Serum albumin $\leq$ 2.8 g/dL
    e. INR $\geq$ 1.7
    f. Direct bilirubin $\geq$ 2.0 mg/dL
5. A MELD score $\geq$ 15 or Child-Pugh-Turcotte Stage B or C.
6. Known positivity for Human Immunodeficiency Virus (HIV) infection.
7. Any patient who had major surgery within 8 weeks of Day 1, significant traumatic injury, or anticipation of need for major surgical procedure during the course of the study.

8. Weight reduction surgery within the past 3 years.

9. Any subject with current, significant alcohol consumption or a history of significant alcohol consumption for a period of more than 3 consecutive months any time within 1 year prior to screening will be excluded. Significant alcohol consumption is defined as more than 20 grams per day in females and more than 30 grams per day in males. On average, a standard drink in the US is considered to be 14 grams of alcohol equivalent to 12 fluid oz of regular beer (5% alcohol), 5 fluid oz of table wine (12% alcohol), or 1.5 fluid oz of 80 proof spirits (40% alcohol).

10. Any subject who has clinically significant and uncontrolled cardiovascular disease (e.g., uncontrolled hypertension, myocardial infarction, unstable angina), New York Heart Association (NYHA) Grade II or greater congestive heart failure, serious cardiac arrhythmia requiring medication, or Grade II or greater peripheral vascular disease within 12 months prior to Day 1.

11. Any subject with concurrent infection including diagnoses of fever of unknown origin (FUO) (subjects must be afebrile at the start of therapy).

12. Any history of malignancy in the previous 5 years in the judgement of the Principal Investigator, except for the following adequately-treated non-metastatic basal cell skin cancer; any other type of skin cancer, except melanoma, that has been adequately treated and has not recurred for at least 1 year prior to enrollment; and adequately treated in situ cervical cancer that has not recurred for at least 1 year prior to enrollment.

13. Participation in an investigational new drug (IND) trial in the 30 days before randomization.

14. Clinically significant medical or psychiatric condition considered a high risk for participation in an investigational study.

15. Failure to give informed consent.

16. Subjects with known allergies to the study drug or any of its excipients.

17. Is an employee or family member of the investigator or study site personnel.

18. Any subject who cannot undergo an MRI, e.g., due to certain metal or electronic device implants, as determined by the Principal Investigator.

4.2 Withdrawal of Subjects From the Study

The duration of the study is defined for each subject as the date signed written informed consent is provided through the last follow-up visit 30 days following the last drug infusion.

4.2.1 Reasons for Withdrawal/Discontinuation

Subjects may withdraw from the study at any time and for any reason without prejudice to their future medical care by the investigator or at the study site. Every effort should be made to keep subjects in the study. The reasons for subjects not completing the study will be recorded. A subject may be withdrawn from the study for any of the following reasons:

1. Significant noncompliance with the protocol.
2. A serious or intolerable adverse event(s) (AE[s]) that in the investigator's opinion requires withdrawal from the study.
3. Laboratory safety assessments that reveal clinically significant hematological or biochemical changes from the baseline values.
4. Symptoms or an intercurrent illness not consistent with the protocol requirements or that justifies withdrawal.
5. Lost to follow-up.
6. Pregnancy.
7. The subject withdraws consent or the investigator or sponsor decide to discontinue the subject's participation in the study.

The investigator will also withdraw a subject if Galectin Therapeutics terminates the study. Upon occurrence of a serious or intolerable AE, the investigator will confer with the sponsor. If a subject is discontinued because of an AE, the event will be followed until it is resolved. Any subject may withdraw his or her consent at any time.

4.2.2 Handling of Withdrawals

Subjects are free to withdraw from the study or study treatment at any time upon request. Subject participation in the study may be stopped at any time at the discretion of the investigator or at the request of the sponsor.

Subjects who discontinue study treatment or active participation in the study will no longer receive study drug. When a subject withdraws from the study, the reason(s) for withdrawal shall be recorded by the investigator on the relevant page of the electronic case report form (eCRF). Whenever possible, all subjects who discontinue study treatment or withdraw from the study prematurely will undergo all end of study assessments. Subjects who fail to return for final assessments will be contacted by the study center (2 documented phone calls followed by 1 registered letter) in an attempt to have them comply with the protocol.

It is vital to obtain follow-up data on any subject withdrawn because of an AE or serious AE (SAE). In every case, efforts must be made to undertake protocol-specified, safety, follow-up procedures.

4.2.3 Replacements

Subjects who are randomly assigned to study drug and prematurely discontinued from the study may be replaced.

5 Study Treatments

Subjects will be randomly assigned to placebo or GR-MD-02, 8 mg/kg lean body weight of GR-MD-02, using a 1:1 allocation ratio. An electronic randomization schedule at CTI will be used to administer the randomization schedule.

5.1 Method of Assigning Subjects to Treatment Groups

GR-MD-02 for Injection is supplied in a sterile aqueous solution of phosphate-buffered saline at a concentration of 30 mg/mL GR-MD-02 and should be diluted to the target dose in normal saline and administered intravenously over a period of approximately 60 minutes. The product is expected to be stable at pH 4.0 to pH 7.5.

GR-MD-02 solution will be supplied in 10-mL vials with the following composition to the study center (Table 5-1).

Table 5-1 Composition of GR-MD-02 Concentrate

Ingredient	Concentration
GR-MD-02	300 mg
USP sodium chloride	82 mg
USP disodium phosphate heptahydrate	14.4 mg
USP monosodium phosphate monohydrate	2.4 mg
USP sterile water for injection	to 10 mL

Abbreviations: USP = United States Pharmacopeia

Instructions on dosing preparation are provided in the Pharmacy Manual.

Once the infusion solutions are prepared, they may be stored at controlled-room temperature and should be administered within 24 hours of preparation. Infusions should be administered intravenously over a period of approximately 60 minutes. Frequency of administration should be once every 2 weeks (bi-weekly) according to the schedule of events (Section 11).

5.2 Identity of Investigational Product

GR-MD-02 (galactoarabino-rhamnogalaturonate) is a soluble polysaccharide composed of an alternating α-(1,2)-L-rhamnosyl-α-(1,4)-D-galacturonosyl backbone with side branches composed of mainly galactose and arabinose oligosaccharides.

GR-MD-02 for injection is a clear, light yellow-tan solution and is supplied in 10-mL sterile vials at a concentration of 30 mg/mL of GR-MD-02 in phosphate buffered saline.

Detailed instructions for the preparation and administration of the GR-MD-02 by slow IV drip over a period of approximately 60 minutes will be provided in the Pharmacy Manual.

Lean body mass (LBM) will be used for dosing because it is anticipated that many subjects will be obese and GR-MD-02 is distributed primarily in the blood compartment. The dose calculated based on the LBM at the first infusion visit will be maintained at each subsequent infusion visit since it is highly unlikely that LBM will vary significantly (e.g. by 10%) during the course of the study. Lean body mass will be estimated from height and weight measurements using formulas that have been well-validated in obese individuals:

- Males: LBM = 9270 x TBW (total body weight) / (6680 + 216 x BMI)
- Females: LBM = 9270 x TBW / (8780 + 244 x BMI)

Where TBW is in kg and BMI is mass (weight in kg)/height (height in $m)^2$. Tables of LBM values for a range of heights and weights will be provided to the pharmacies participating in the study as a check for the calculations.

The study pharmacist at the study center will be unblinded in the preparation of IMP for infusion. Infusion solutions will be prepared as specified in the Pharmacy Manual in IV bags and placed in amber colored IV bag covers with amber colored tubing and infusion sets. The amber colored bags will be sealed with tamper evident tapes. Once prepared by the unblinded pharmacist, the placebo and drug solutions in the IV infusion set up will be indistinguishable. Thus, the study subjects, primary investigators, and medical personnel will be blinded throughout the study as to whether the subject is receiving active drug or placebo. Upon completion of IMP infusion, documentation of the discarded infusion set must be recorded to indicate no unblinding occurred.

5.3 Management of Clinical Supplies

5.3.1 Study Drug Packaging and Storage

Galectin Therapeutics will provide study drug to the study centers. The following drug supplies will be used in the study:

- GR-MD-02, supplied as 10-mL sterile vials
- Placebo (phosphate-buffered saline solution), supplied as 10-mL sterile vials

GR-MD-02 for Injection (30 mg/mL) and placebo to be used for the study (phosphate buffered saline) in 10-mL vials are manufactured by Catalent Pharma Solutions, Woodstock, Illinois, and packaged and labeled for the clinical study by Catalent Pharma Solutions, Philadelphia, Pennsylvania.

The GR-MD-02 for Injection (30 mg/mL) vials and placebo vials are to be stored under refrigerated conditions at 2°C to 8°C (35° to 46°F) until preparation of the drug infusion solution. To prepare the final infusion solution for administration, the GR-MD-02 will be diluted in sterile normal saline as required to achieve the target dose. Instructions on dosing preparation are provided in the Pharmacy Manual.

5.3.2 Test Article Accountability

The investigator and pharmacy will maintain accurate records of receipt of all test articles, including dates of receipt. In addition, accurate records will be kept regarding when and how much test article is dispensed and used by each subject in the study. Reasons for departure from the expected dispensing regimen must also be recorded. Upon completion of drug infusion, documentation of the discarded infusion set must be recorded to indicate no unblinding occurred. At the completion of the study, to satisfy regulatory requirements regarding drug accountability, study drug will be reconciled and retained or destroyed according to applicable regulations.

5.3.3 Other Supplies

In addition to drug and placebo for the study, the study center will also be provided sterile syringe filters, amber colored bag covers (with tamper evident tape) and amber colored

infusion tubing sets. See the Pharmacy Manual for the specifications and details regarding these ancillary supplies.

5.4 Overdose Management

An overdose is any dose of study treatment given to a subject or taken by a subject that exceeds the dose described in the protocol. Any overdose, with or without associated AEs, must be promptly reported to the managing CRO, CTI and Galectin Therapeutics.

- **SAE Hotline: 1-877-755-0742**
- **SAE eFax line: 1-800-725-0933**
- **eFax email: ctisafety@ctifacts.com**

Overdoses without signs or symptoms do not need to be recorded as AEs; in case of any AEs associated with the overdose, these should be reported on relevant AE/SAE sections in the CRF.

5.4.1 Treatment of Overdose

There are no known or anticipated adverse effects of GR-MD-02 overdose. The subject should be observed, but there are no specific therapies to be instituted.

5.4.2 Medication Errors

A medication error is any unintentional error in prescribing, dispensing, or administration of the study drug. The study drug is being prepared and administered at the study center by trained personnel, thereby reducing the risk of medication errors.

Acceptable treatment windows for infusions will be ± 3 days. Infusions given outside of accepted windows would be considered “out of window” doses. If a dose is out of window, the subject should be brought back into compliance with their visit dosing schedule. The subject should not dose within 7 days of the previous or next dose unless the medical monitor is consulted.

Any AEs resulting from medication errors will be recorded as AEs in the CRF.

5.4.3 Treatment of Medication Errors

Should a medication error occur, the subject should be observed; but there are no specific therapies to be instituted.

5.5 Misuse for Illegal Purposes

GR-MD-02 does not have known effects that may be addictive and there are no other reasons for misuse.

5.6 Blinding

The aqueous solution of GR-MD-02 is a light yellow-tan color. It has not been possible to match the color with a placebo formulation. Therefore, the study pharmacist at each study center will be unblinded in the preparation of IMP for infusion. The formulated 10-mL solutions will be prepared for infusion as specified in the Pharmacy Manual in IV bags and placed in amber colored IV bag covers with amber colored tubing and infusion sets. The amber colored bags will be sealed with tamper evident tape. Placebo and drug solutions to be administered in the IV infusion set up are indistinguishable. Thus, the study subjects, primary investigators, and medical personnel will be blinded to whether the subject is receiving study drug. Infusion of the study drug causes no local reaction in animal studies or in humans participating in the Phase 1 clinical study and, therefore should not affect blinding.

5.6.1 Breaking the Blind

A subject’s treatment assignment will not be broken until the end of the study unless medical treatment of the subject depends on knowing the study treatment the subject received. In the event that the blind needs to be broken because of a medical emergency, the investigator may unblind an individual subject’s treatment allocation. As soon as possible, the investigator should first contact the medical monitor to discuss the medical emergency and the reason for revealing the actual treatment received by that subject. The treatment assignment will be unblinded by the investigator through the blinding code held by CTI. Reasons for treatment unblinding must be clearly explained and justified in the CRF. The date on which the code was broken together with the identity of the person responsible must also be documented.

5.7 Treatment Compliance

Subject compliance will be determined by infusion records.

Acceptable windows for study drug dosing are ± 3 days. Infusions given outside of accepted windows would be considered "out of window" doses. If a dose is out of window, the subject should be brought back into compliance with their visit dosing schedule. The subject should not dose within 7 days of the previous or next dose unless the medical monitor is consulted.

5.8 Prior and Concomitant Therapy

Use of all concomitant medications will be recorded in the subject's CRF. The minimum requirement is that drug name, reason for use, dose, and the dates of administration are to be recorded. This will include all prescription drugs, herbal products, vitamins, minerals, and over-the-counter (OTC) medications. Any changes in concomitant medications also will be recorded in the subject's CRF.

All prior treatments for liver cirrhosis and NASH will be recorded in the CRF. All other concomitant medications taken from 30 days prior to screening and throughout the entire duration of the subject's time on study, will be collected in the CRF. Concomitant medications are defined as medications taken any time after the start of exposure to IMP. Prior medications are defined as medications discontinued prior to the start of exposure to study drug.

Any concomitant medication deemed necessary for the welfare of the subject during the study may be given at the discretion of the investigator. It is the responsibility of the investigator to ensure that details regarding the medication are recorded in full in the CRF.

5.9 Prohibited Medications or Therapies

None.

6 Study Assessments and Procedures

Before any study procedures are performed, all potential subjects will sign an ICF. Subjects will have the opportunity to have any questions answered before signing the ICF. The investigator must address all questions raised by the subject. The investigator will also sign the ICF.

The schedule of events for randomization, treatment phase, and follow-up in section 11.

6.1 Study Visits

6.1.1 Screening Visit (Week -6 to Day 0)

Patients with a liver biopsy with NASH with Brunt Stage 3 fibrosis may enter screening. The screening visit window is up to 6 weeks prior to randomization. Subjects who fail to meet eligibility criteria within this 6-week period due to an abnormal laboratory result may undergo retesting of the abnormal laboratory parameter during the screening window and at the discretion of the investigator and with prior approval of the medical monitor. During the screening visit, the following information will be collected:

- Inclusion/exclusion criteria
- Medical, surgical, and medication history (concomitant medications taken from 30 days prior to screening)
- Collect demographic information
- 12-lead ECG
- Hematology
- Blood chemistry, including alpha-2 macroglobulin
- Coagulation profile
- Viral hepatitis B and C serology

- HIV serology
- Urinalysis
- Serum pregnancy test (females of childbearing potential only)
- Complete physical examination (including height [in cm] and weight [in kg]) with particular attention to examination for stigmata of liver disease/cirrhosis
- Vital sign measurements (including heart and respiratory rate, blood pressure, and body temperature)
- AE monitoring

6.1.2 Treatment Phase

6.1.2.1 Randomization

Randomization must occur within 6 weeks after screening has begun.

Prior to infusion Visit 1, subjects who meet the inclusion and exclusion criteria will be randomly assigned (1:1) placebo or GR-MD-02 in a dose of 8 mg/kg lean body mass.

6.1.2.2 Two Weeks Prior to Infusion Visit 1

The following will be conducted within 2 weeks prior to the first infusion:

- LiverMultiScan
- MR-Elastography
- FibroScan

6.1.2.3 Infusion Visit 1 (Week 1), Infusion Visit 2 (Week 3 ± 3 days), Infusion Visit 3 (Week 5 ± 3 days), Infusion Visit 4 (Week 7 ± 3 days), Infusion Visit 5 (Week 9 ± 3 days), Infusion Visit 6 (Week 11 ± 3 days), Infusion Visit 7 (Week 13 ± 3 days), Infusion Visit 8 (Week 15 ± 3 days), Infusion Visit 9 (Week 17 ± 3 days)

The following procedures and assessments will be conducted prior to administration of study drug:

- Limited physical examination (including weight, heart, lung and abdominal examination)
- Vital sign measurements (including heart and respiratory rate, blood pressure, and body temperature)
- Hematology, blood chemistry-including alpha-2 macroglobulin, and urine pregnancy test (Visit 1, 5 and 9 only)
- ECG (Visit 9 only)

The following information will be collected after study drug administration:

- AE monitoring
- Concomitant medications

6.1.3 Follow-up study evaluations (7-21 Days after Final Dose)

Within 7 to 21 days after the final 9th dose of IMP or where feasible following study termination, the following information will be collected:

- LiverMultiScan
- MR-Elastography
- FibroScan

6.1.4 Follow-up/Early Termination

30 days after the last infusion visit the following information will be collected:

- Limited physical examination (including weight, heart, lung and abdominal examination)
- Vital sign measurements (including heart and respiratory rate, blood pressure, and body temperature)
- AE monitoring
- Concomitant medications

6.2 Efficacy Assessments

6.2.1 LiverMultiScan

LiverMultiScan® is a new method developed by Perspectum Diagnostics™ which is a novel magnetic resonance (MR) protocol that can be performed without intra-venous contrast on existing scanners. MR methods are ideally suited for tissue characterization as they can sample the entire liver quickly, and are safe, reproducible, and widely available. Using multiparametric MR, investigators were able to objectively quantify hepatic fibrosis, steatosis and hemosiderosis, an important step towards a safer alternative to liver biopsy (9). MR data will be evaluated by Perspectum Diagnostics™ and the cT1 generated as the primary endpoint for evaluation, which correlates with the degree of inflammation and fibrosis in the liver. LMS will be obtained following at least a 6 hour fast, including water.

6.2.2 MR Elastography

MR elastography is a method for evaluating the stiffness of the entire liver using an electromechanical pulse and magnetic resonance imaging, and has been demonstrated to be useful in the evaluation of NASH with advanced fibrosis (8). MRE will be obtained at the same time as LMS. The procedure will be performed on Siemans 1.5 Tesla X 2 and 3.0 x 1 machines under the direction of Dr. Christopher J. Lisanti, an experienced radiologist. Blinded paired MR-E exams from prior to randomization and at the end of the study will be evaluated Dr. Lisanti.

6.2.3 FibroScan

FibroScan is an FDA approved, non-invasive diagnostic instrument that uses an electromechanical vibrator and pulse-echo ultrasound to evaluate the elastic shear wave in liver tissue which is a measure of liver stiffness. The stiffness of the liver is recorded as the pressure measurement of kiloPascals. The stiffness of the liver correlates with the degree of liver fibrosis as assessed by liver biopsy, including subjects with NASH (10). One advantage of this method is that the volume of liver tissue assessed is ~100-times greater than volume assessed by liver biopsy. As such FibroScan represents a promising non-invasive, outpatient method for measuring changes in liver fibrosis over time. FibroScan evaluations will be performed after an 8 hour fast according to the manufacturer's instructions.

6.3 Safety Assessments

All subjects receiving any part of at least 1 infusion of study treatment will be evaluated for safety. Safety assessments will include incidence of AEs during study treatment, emergent physical examination abnormalities, emergent vital sign and ECG abnormalities, and laboratory parameter abnormalities.

6.3.1 Vital Signs

Vital sign measurements (including heart and respiratory rate, blood pressure and body temperature) will be collected before administration of IMP at time points specified in the schedule of assessments. Blood pressure will be obtained with the subject in the supine position and measured twice consecutively with a 1-minute interval between measurements. The average of the 2 measurements will be recorded.

6.3.2 Physical Examination

A complete physical examination, including height and weight, will be performed at screening, with particular attention to examination for stigmata of liver disease/cirrhosis. A limited physical examination will only include weight, heart, lung, and abdominal examination according to the time points specified in the schedule of assessments.

6.3.3 Electrocardiogram

A standard 12-lead ECG will be performed according to the schedule of events. Twelve-lead ECGs will be systematically digitally recorded after the subject has been in the supine position for at least 10 minutes. The electrodes will be positioned in the same location for each ECG recording. Electrocardiograms will be recorded before blood sampling.

6.3.4 Adverse Events

6.3.4.1 Definitions of Adverse Events

The investigator is responsible for reporting all treatment-emergent AEs (TEAEs) that are observed or reported during the study, regardless of their relationship to IMP or their clinical significance.

An AE is defined as any untoward medical occurrence in a subject enrolled into this study regardless of its causal relationship to IMP. Subjects will be instructed to contact the investigator at any time after randomization if any symptoms develop.

A TEAE is defined as any event not present before exposure to IMP or any event already present that worsens in either intensity or frequency after exposure to IMP.

An SAE is defined as any event that results in death, is immediately life threatening, requires inpatient hospitalization or prolongation of existing hospitalization, results in persistent or significant disability/incapacity, or is a congenital anomaly/birth defect. Important medical events that may not result in death, be life threatening, or require hospitalization may be considered SAEs when, based upon appropriate medical judgment, they may jeopardize the subject or may require medical or surgical intervention to prevent one of the outcomes listed in this definition. Examples of such medical events include allergic bronchospasm requiring intensive treatment in an emergency room or at home, blood dyscrasias or convulsions that do not result in inpatient hospitalization, or the development of drug dependency or drug abuse.

6.3.4.2 Eliciting and Documenting Adverse Events

All AEs (serious and non-serious) will be assessed beginning at screening and up to 42 days after the last dose of IMP.

Serious AEs that occur more than 42 days after the last dose of IMP need not be reported unless the investigator considers them related to IMP.

At every study visit, subjects will be asked a standard nonleading question to elicit any medically related changes in their well-being. They will also be asked if they have been hospitalized, had any accidents, used any new medications, or changed concomitant medication regimens (both prescription and OTC medications).

In addition to subject observations, AEs identified from any study data (e.g., laboratory values, physical examination findings, ECG changes) or identified from review of other documents that are relevant to subject safety will be documented on the AE page in the CRF.

6.3.4.3 Reporting Adverse Events

All AEs reported or observed during the study will be recorded on the AE page in the CRF. Information to be collected includes drug treatment, dose, event term, time of onset, investigator-specified assessment of severity and relationship to IMP, time of resolution of the event, seriousness, any required treatment or evaluations, and outcome. Adverse events resulting from concurrent illnesses, reactions to concurrent illnesses, reactions to concurrent medications, or progression of disease states must also be reported. All AEs will be followed to adequate resolution. The Medical Dictionary for Regulatory Activities (MedDRA, Version 17.1) will be used to code all AEs.

Any medical condition that is present at the time that the subject is screened but does not deteriorate should not be reported as an AE. However, if it deteriorates at any time during the study, it should be recorded as an AE.

Any AE that meets SAE criteria (Section 6.3.4.1) must be reported to the sponsor (designee) immediately (i.e., within 24 hours) after the time study center personnel first learn about the event.

Study personnel should complete the AE CRF, designating the event as serious.

6.3.4.4 Assessment of Severity

The severity, or intensity, of an AE refers to the extent to which an AE affects the subject's daily activities. The intensity of the AE will be rated as mild, moderate, or severe using the following criteria:

Mild:	These events require minimal or no treatment and do not interfere with the subject's daily activities.
Moderate:	These events result in a low level of inconvenience or concern with the therapeutic measures. Moderate events may cause some interference with normal functioning.
Severe:	These events interrupt a subject's usual daily activity and may require systemic drug therapy or other treatment. Severe events are usually incapacitating.

Changes in the severity of an AE should be documented to allow an assessment of the duration of the event at each level of intensity to be performed. Adverse events characterized as intermittent do not require documentation of onset and duration of each episode.

6.3.4.5 Assessment of Causality

The investigator's assessment of an AE's relationship to IMP is part of the documentation process, but it is not a factor in determining what is or is not reported in the study. If there is any doubt as to whether a clinical observation is an AE, the event should be reported.

The relationship or association of the test article in causing or contributing to the AE will be characterized using the following classification and criteria:

<u>Unrelated:</u> This relationship suggests that there is no association between the IMP and the reported event.

<u>Possible:</u> This relationship suggests that treatment with the IMP caused or contributed to the AE, ie, the event follows a reasonable temporal sequence from the time of drug administration or follows a known response pattern to the IMP, but could also have been produced by other factors.

<u>Probable:</u> This relationship suggests that a reasonable temporal sequence of the event with drug administration exists and, based upon the known pharmacological action of the drug, known or previously reported adverse reactions to the drug or class of drugs, or judgment based on the investigator's clinical experience, the association of the event with the IMP seems likely. The event disappears or decreases on cessation or reduction of the dose of IMP.

<u>Definite:</u> This relationship suggests that a definite causal relationship exists between drug administration and the AE, and other conditions (concurrent illness, progression/expression of disease state, or concurrent medication reaction) do not appear to explain the event. The event reappears or worsens if the IMP is re-administered.

6.3.4.6 Exceptions

While the following events may fulfill the serious criteria, they do not need to be reported as SAEs: hospitalizations/emergency room stays lasting less than 24 hours not meeting other serious criteria, hospitalizations for baseline conditions that do not worsen after starting study participation, hospitalizations for elective procedures anticipated or scheduled prior to study participation, and events expected as part of progression of disease under study.

6.3.4.7 Follow-Up of Subjects Reporting Adverse Events

All AEs must be reported in detail on the appropriate page in the CRF and followed to satisfactory resolution, until the investigator deems the event to be chronic or not clinically significant, or until the subject is considered to be stable.

6.3.5 Pregnancy

Pregnancy is not regarded as an AE unless there is a suspicion that an IMP may have interfered with the effectiveness of a contraceptive medication. Any pregnancy that occurs during study participation must be reported using a clinical study pregnancy form. The pregnancy must be followed up to determine outcome (including spontaneous miscarriage, elective termination, normal birth, or congenital abnormality) and status of mother and child,

even if the subject was discontinued from the study. Pregnancy complications and elective terminations for medical reasons must be reported as an AE or SAE. Spontaneous miscarriages must be reported as an SAE.

Pregnancy must be reported in the same manner as an SAE to CTI pharmacovigilance (PVG) by the study center within 24 hours of being informed of the event. If a pregnancy occurs during the study, study medication must be discontinued immediately (or per sponsor directive), and the pregnancy report form submitted to CTI PVG via the safety fax line. The study center should submit the pregnancy follow-up form to document the outcome of the pregnancy (health of the neonate). In the event of a miscarriage, therapeutic abortion, death *in utero*, or the pregnancy outcome leads to an SAE for the mother, follow the Procedure for Reporting an SAE. In the event of a congenital anomaly, an SAE form for the baby must be completed.

Any SAE occurring in association with a pregnancy that is brought to the investigator's attention after the subject has completed the study and considered by the investigator as possibly related to the study treatment must be promptly reported to Galectin Therapeutics Inc.

6.3.6 Laboratory Analyses

Any abnormal laboratory test results (hematology, clinical chemistry, or urinalysis) or other safety assessments (eg, ECGs, radiological scans, vital sign measurements), including those that worsen from baseline, believed to be clinically significant in the medical and scientific judgment of the investigator are to be recorded as AEs or SAEs.

However, any clinically significant laboratory assessments that are associated with the underlying disease, unless judged by the investigator to be more severe than expected for the subject's condition, are not to be reported as AEs or SAEs.

6.3.7 Hematology

Tests will include the following:

Hemoglobin (Hgb)	Differential:
Hematocrit (Hct)	Neutrophils
Red blood cells (RBCs)	Lymphocytes
White blood cells (WBCs)	Monocytes
Red cell indices	Basophils
Platelet count	Eosinophils
	Prothrombin time
	Partial thromboplastin time
	Haptoglobin

6.3.8 Blood Chemistry

Tests will include the following:

Alanine aminotransferase (ALT)	Calcium	Magnesium
Albumin	Chloride	Phosphorus
Alkaline phosphatase	Creatinine	Potassium
alpha-2 macroglobulin	Fasting insulin level	Sodium
Aspartate aminotransferase (AST)	Gamma-glutamyl transferase (GGT)	Total and direct bilirubin
Bicarbonate	Glucose	Total protein
Blood urea nitrogen (BUN)	Lactate dehydrogenase (LDH)	Uric acid

6.3.9 Urinalysis

Refer to the Study Manual for collecting urinalysis samples. Tests will include the following:

Color	Glucose	RBC
Clarity	Blood	Hyaline and other casts
pH	Bilirubin	Bacteria
Specific gravity	Leukocyte esterase	Epithelial cells
Ketones	Nitrite	Crystals
Protein	WBC	Yeast
		Mucus

6.4 Sample Collections

All safety laboratory tests will be collected after the subject has been fasting for at least 8 hours. Each subject with have blood drawn for hematology and chemistry (including fasting insulin level and alpha-2 macroglobulin).

7 Statistical and Analytical Plan

A summary of statistical methods is presented in the following sections and will be described in more detail in the Statistical and Analysis Plan.

7.1 Primary Efficacy Endpoint

The primary efficacy endpoint analysis is the baseline-adjusted change in cT1 as assessed by LMS at 17-19 weeks in subjects treated with placebo as compared to subjects treated with GR-MD-02 (8 mg/kg/week).

7.2 Secondary Efficacy Endpoints

The secondary endpoints are:

- The baseline-adjusted change in liver stiffness as determined by MRE at week 17-19.
- The baseline-adjusted change in liver stiffness as determined by FibroScan® Score at week 17-19.

7.3 Exploratory Endpoints

An exploratory objective will be to evaluate the correlation of the three diagnostic modalities of LMS, MRE, and FS. Additionally, serum will be analyzed for alpha-2 macroglobulin.

7.4 Safety Endpoints

The safety endpoints include the incidence of AEs during study treatment, emergent physical examination abnormalities, emergent vital sign and ECG abnormalities, and laboratory parameter abnormalities.

7.5 Sample Size Calculations

A total of approximately 30 subjects will be enrolled in the study. Sample size calculations are based on the comparison of the primary efficacy variable, change in cT1 as assessed by LMS from baseline, with the following assumptions:

1. Mean baseline cT1 in treatment groups, 950 ms

2. True mean change in cT1 as measured by LMS from baseline at 17-19 weeks in the placebo group, $\Delta p = 0$

3. True mean change in cT1 as measured by LMS from baseline at 17-19 weeks in GR-MD-02 dose group, $\Delta G = 45.1$ ms (milliseconds)

4. Common standard deviation for difference in cT1, $\sigma = 42.5$ ms

5. Null hypothesis, H_0: $\theta = G - \Delta p = 0$

6. Type I error, $\alpha = 0.05$ (2-sided significance test)

7. Power = 80%

8. Statistical test = 2-sample t-test for mean difference

9. Randomization ratio = 1:1

7.6 Analysis Sets

The following analysis sets will be used in the statistical analyses.

Full-analysis set (FAS): The FAS, or intent-to-treat group (ITT), will consist of all subjects who were randomly assigned to study drug. All analyses using the FAS will group subjects according to randomized treatment.

Modified-Intent-to-Treat (mITT) analysis set: The mITT will consist of all subjects who were randomized, received at least 1 infusion, and had at least one post-baseline efficacy assessment. All analyses using the mITT will group subjects according to randomized treatment.

Per-protocol set (PPS): The PPS will consist of all FAS subjects who have at least 80% compliance with study treatment, have not taken any prohibited medication, have no significant protocol deviations, and restricted to each subject’s time on study drug plus 30 days thereafter for the analysis of progression of efficacy events. All analyses using the PPS will group subjects according to treatment actually received.

Safety set: The safety set will consist of all subjects who received any study drug. All analyses using the safety set will group subjects according to treatment actually received.

The FAS will be used as the primary efficacy analysis set.

7.7 Description of Subgroups to be Analyzed

No subgroup analyses are planned.

7.8 Statistical Analysis Methodology

Statistical analysis will be performed using SAS software Version 9.1.3 or later. Continuous variables will be summarized by randomized treatment group using the mean, the standard deviation, median, 25th and 75th percentile, minimum value, and maximum value. Point estimates and the associated 95% confidence interval (CI) for the difference in baseline-adjusted efficacy parameters will be summarized in a similar manner. Categorical variables will be summarized by randomization treatment group using frequency counts and percentages. All baseline and post-baseline data used for safety and efficacy evaluations will be listed in data listings. All statistical tests will be 2-sided and will be at the 5% level of significance.

For analysis variables that are not normally distributed, alternative methods will be applied including the use of the Poisson-regression model (or the negative binomial) for counts data. For others, a nonparametric method based on Hodge-Lehmann estimate (or similar) and associated 95% CI on median difference will be applied.

The primary method of analysis will use the last observation carried forward (LOCF) method to impute missing post-baseline data.

Details of the statistical analyses, methods, adjustments for multiplicity, and data conventions will be described in the SAP.

7.8.1 Analysis of Primary Efficacy Endpoint

The primary efficacy endpoint analysis is the baseline-adjusted change in cT1 as assessed by LMS at 17-19 weeks in subjects treated with placebo as compared to subjects treated with GR-MD-02 (8 mg/kg/week). Change in cT1 as assessed by LMS from baseline will be

compared between treatments groups using analysis of covariance (ANCOVA) adjusted for baseline cT1 as assessed by LMS. Treatment group will be included in the model as indicator variables where T1 = 1 if subject is randomly assigned to GR-MD-02 and 0 otherwise. The following hypothesis will be tested based on the parameter estimates (and standard errors) from the fit of the ANCOVA model:

H01: $\Delta T1 - \Delta P = 0$ vs. HA1: $\Delta T1 - \Delta P \neq 0$

7.8.2 Analysis of Key Secondary Efficacy Endpoints

Key secondary endpoints will be examined using a gatekeeper statistical approach. If the primary endpoint is significant (α <0.05), analysis will proceed to the secondary endpoints. Since the evaluation of secondary endpoints will proceed using a pre-specified hierarchy without statistical correction for multiplicity, if a secondary endpoint does not meet statistical significance, all subsequent analysis will be treated as exploratory only.

7.8.3 Analysis of the Exploratory Efficacy Endpoint

Exploratory endpoints will be analyzed in the same fashion as the primary endpoint.

7.8.4 Safety Analyses

All subjects receiving any part of at least 1 infusion of study treatment will be evaluated for safety. The safety analyses will include evaluation of the incidence of treatment-emergent adverse events (TEAEs), Grade 3 or greater AEs, SAEs and AEs leading to discontinuation of study treatment using the Common Terminology Criteria for Adverse Events Version 4.0 or higher. Laboratory and vital signs assessments will be evaluated over time on study using descriptive statistics. Shift analyses of relevant clinical laboratory parameters will be produced showing shifts across low, normal, and high categories.

All heart rate (bpm), 12-lead ECG parameters, PR Interval (msec), QRS Interval (msec), QT Interval (msec), and QTc Interval (msec) will be measured, and overall interpretation will be summarized for all subjects by study visit including the last visit of the study.

Electrocardiogram parameters will be summarized using descriptive statistics. Mean and mean change from baseline values will be presented for every scheduled assessment. Change

from baseline will be calculated as the post-baseline measurement minus the baseline measurement. If either the baseline or post-baseline value is missing, the observation will not be included in the change from baseline summary. In addition, counts and percentages for ECG overall interpretation (normal, abnormal, clinically significant, and not clinically significant) will be presented for each scheduled assessment.

For physical examinations, the results (normal, abnormal, or not done) by body system of the full physical examination at the randomization visit and follow-up visits will be summarized with descriptive statistics by treatment group and visit. For physical examinations performed at postrandomization time points, the number and percent of subjects with no change or any significant changes since the previous examination will be presented by treatment received. In addition, a shift table will be included to summarize the number and percent of subjects with changes from baseline to each postrandomization visit by body system for each treatment group. The number and percent of subjects with normal and abnormal results (clinically significant vs. not clinically significant) for each body system will be presented by treatment group.

Vital sign measurements (respiration, heart rate, temperature, systolic and diastolic blood pressure), height (at screening only), and BMI will be summarized descriptively at each scheduled visit. Mean and mean change from baseline values will be presented. Change from baseline will be calculated as post-baseline measurement minus baseline measurement. If either the baseline or post-baseline value is missing, the observation will not be included in the change from baseline summary.

All safety data will be listed by subject, parameter, and time point.

7.8.5 Other Analyses

Summary statistical analyses will be provided for baseline assessments such as demographics and medical history.

The numbers and percentages of subjects in each treatment group taking concomitant medications, defined as non-study medications with a stop date on or after the date of the first infusion of study medication, will be summarized by dictionary coded terms (World Health Organization Drug Dictionary (Version 1 Sep 2014). Medications that started prior to the first infusion of study medication but continued during treatment will also be defined as

concomitant. Ongoing medications without stop dates are considered concomitant. Prior medications, defined as nonstudy medications with a stop date before the first infusion of study medication, will also be summarized by treatment group. Medications with partial onset/stop dates that indicate that the medication could be concomitant in relation to the start date of study medication will be classified as concomitant. Otherwise, they will be classified as prior medications.

Investigational medicinal product exposure and overall percent compliance will be calculated per subject in the FAS population and summarized by treatment group using descriptive statistics.

7.8.6 Interim Analyses

There is no planned interim analysis.

7.9 Data Quality Assurance

The sponsor's (or an authorized representative's) Quality Assurance department may conduct on-site audits of all aspects of the clinical study either during the study or after the study has been completed.

The clinical study may also be subject to inspection by regulatory authorities (national or foreign) as well as the IECs/IRBs to ascertain that the study is being or has been conducted in accordance with protocol requirements, GCPs, as well as the applicable regulatory requirements.

7.9.1 Data Management

As part of the responsibilities assumed by participating in the study, the investigator agrees to maintain adequate case histories for the subjects treated as part of the research under this protocol. The investigator agrees to maintain accurate CRFs and source documentation as part of the case histories. These source documents may include hospital records, clinical and office charts, chest x-ray and interpretation, questionnaires, pharmacy dispensing and other records, etc.

Clinical data management will be performed in accordance with applicable Galectin Therapeutics standards and data cleaning procedures to ensure the integrity of the data, e.g., removing errors and inconsistencies in the data. Adverse events and concomitant medication terms will be coded using the MedDRA, an internal validated medication dictionary.

8 Ethics

8.1 Independent Ethics Committee or Institutional Review Board

Federal regulations and the International Conference on Harmonisation (ICH) guidelines require that approval be obtained from an Institutional Review Board (IRB)/ Independent Ethics Committee (IEC) before participation of human subjects in research studies. Before study onset, the protocol, informed consent, advertisements to be used for the recruitment of study subjects, and any other written information regarding this study to be provided to the subject or the subject's legal guardian must be approved by the IRB/IEC. Documentation of all IRB/IEC approvals and of the IRB/IEC compliance with ICH harmonised tripartite guideline E6(R1): Good Clinical Practice (GCP) will be maintained by the study center and will be available for review by the sponsor or its designee.

All IRB/IEC approvals should be signed by the IRB/IEC chairman or designee and must identify the IRB/IEC name and address, the clinical protocol by title or protocol number or both, and the date approval or a favorable opinion was granted.

The investigator is responsible for providing written summaries of the progress and status of the study at intervals not exceeding 1 year or otherwise specified by the IRB/IEC. The investigator must promptly supply the sponsor or its designee, the IRB/IEC, and, where applicable, the institution, with written reports on any changes significantly affecting the conduct of the study or increasing the risk to subjects.

8.2 Ethical Conduct of the Study

The study will be performed in accordance with the ethical principles that have their origin in the Declaration of Helsinki, ICH Good Clinical Practice, and all applicable regulations.

8.3 Subject Information and Consent

A written informed consent in compliance with US Title 21 Code of Federal Regulations (CFR) Part 50 shall be obtained from each subject before entering the study or performing any unusual or non-routine procedure that involves risk to the subject. An informed consent template may be provided by the sponsor to investigative study centers. If any institution-specific modifications to study-related procedures are proposed or made by the study center,

the consent should be reviewed by the sponsor or its designee or both before IRB/IEC submission. Once reviewed, the consent will be submitted by the investigator to his or her IRB/IEC for review and approval before the start of the study. If the ICF is revised during the course of the study, all active participating subjects must sign the revised form.

Before recruitment and enrollment, each prospective subject or his or her legal guardian will be given a full explanation of the study and be allowed to read the approved ICF. Once the investigator is assured that the subject/legal guardian understands the implications of participating in the study, the subject/legal guardian will be asked to give consent to participate in the study by signing the ICF.

The investigator shall retain the signed original ICF(s) and give a copy of the signed original form to the subject or legal guardian.

9 Investigator's Obligations

The following administrative items are meant to guide the investigator in the conduct of the study but may be subject to change based on industry and government standard operating procedures, working practice documents, or guidelines. Changes will be reported to the IRB/IEC but will not result in protocol amendments.

9.1 Confidentiality

All laboratory specimens, evaluation forms, reports, and other records will be identified in a manner designed to maintain subject confidentiality. All records will be kept in a secure storage area with limited access. Clinical information will not be released without the written permission of the subject (or the subject's legal guardian), except as necessary for monitoring and auditing by the sponsor, its designee, the US FDA, or the IRB/IEC.

The investigator and all employees and coworkers involved with this study may not disclose or use for any purpose other than performance of the study any data, record, or other unpublished, confidential information disclosed to those individuals for the purpose of the study. Prior written agreement from the sponsor or its designee must be obtained for the disclosure of any said confidential information to other parties.

9.2 Financial Disclosure and Obligations

Investigators are required to provide financial disclosure information to allow the sponsor to submit the complete and accurate certification or disclosure statements required under 21 CFR 54. In addition, the investigator must provide to the sponsor a commitment to promptly update this information if any relevant changes occur during the course of the investigation and for 1 year following the completion of the study.

Neither the sponsor nor CTI is financially responsible for further testing or treatment of any medical condition that may be detected during the screening process. In addition, in the absence of specific arrangements, neither the sponsor nor CTI is financially responsible for further treatment of the subject's disease.

9.3 Investigator Documentation

Prior to beginning the study, the investigator will be asked to comply with ICH E6(R1) 8.2 and Title 21 of the CFR by providing the following essential documents, including but not limited to:

- IRB/IEC approval
- Original investigator-signed investigator agreement page of the protocol
- Form FDA 1572, fully executed, and all updates on a new fully executed Form FDA 1572
- Curriculum vitae for the investigator and each sub-investigator listed on Form FDA 1572
- Financial disclosure information to allow the sponsor to submit complete and accurate certification or disclosure statements required under 21 CFR 54. In addition, the investigators must provide to the sponsor a commitment to promptly update this information if any relevant changes occur during the course of the investigation and for 1 year after the completion of the study.
- IRB/IEC-approved informed consent, samples of study center advertisements for recruitment for this study, and any other written information regarding this study that is to be provided to the subject or legal guardian, and

9.4 Study Conduct

The investigator agrees that the study will be conducted according to the principles of ICH E6(R1). The investigator will conduct all aspects of this study in accordance with all national, state, and local laws or regulations. Study information from this protocol will be posted on publicly available clinical trial registers before enrollment of subjects begins.

9.5 Adherence to Protocol

The investigator agrees to conduct the study as outlined in this protocol in accordance with ICH E6(R1) and all applicable guidelines and regulations.

9.6 Adverse Events and Study Report Requirements

By participating in this study the investigator agrees to submit reports of SAEs according to the time line and method outlined in the protocol. In addition, the investigator agrees to submit annual reports to the study center IRB/IEC as appropriate.

9.7 Investigator's Final Report

Upon completion of the study, the investigator, where applicable, should inform the institution; the investigator/institution should provide the IRB/IEC with a summary of the study's outcome and the sponsor and regulatory authority(ies) with any reports required.

9.8 Records Retention

Essential documents should be retained until at least 2 years after the last approval of a marketing application in an ICH region and until there are no pending or contemplated marketing applications in an ICH region or at least 2 years have elapsed since the formal discontinuation of clinical development of the IMP. These documents should be retained for a longer period, however, if required by the applicable regulatory requirements or by an agreement with the sponsor. It is the responsibility of the sponsor to inform the investigator/institution as to when these documents no longer need to be retained.

9.9 Publications

After completion of the study, the data may be considered for reporting at a scientific meeting or for publication in a scientific journal. In these cases, the sponsor will be responsible for these activities and will work with the investigators to determine how the manuscript is written and edited, the number and order of authors, the publication to which it will be submitted, and other related issues. The sponsor has final approval authority over all such issues.

Data are the property of the sponsor and cannot be published without prior authorization from the sponsor, but data and publication thereof will not be unduly withheld.

10 Study Management

10.1 Monitoring

10.1.1 Monitoring of the Study

The clinical monitor, as a representative of the sponsor, has the obligation to follow the study closely. In doing so, the monitor will visit the investigator and study center at periodic intervals, in addition to maintaining necessary telephone and letter contact. The monitor will maintain current personal knowledge of the study through observation, review of study records and source documentation, and discussion of the conduct of the study with the investigator and personnel.

All aspects of the study will be carefully monitored, by the sponsor or its designee, for compliance with applicable government regulation with respect to current GCP and current standard operating procedures.

For the purposes of this study, a blinded and unblinded monitoring team will be used.

10.1.2 Inspection of Records

Investigators and institutions involved in the study will permit study-related monitoring, audits, IRB/IEC review, and regulatory inspections by providing direct access to all study records. In the event of an audit, the investigator agrees to allow the sponsor, representatives of the sponsor, or a regulatory agency (eg, FDA or other regulatory agency) access to all study records.

The investigator should promptly notify the sponsor and CTI of any audits scheduled by any regulatory authorities and promptly forward copies of any audit reports received to the sponsor.

10.2 Management of Protocol Amendments and Deviations

10.2.1 Modification of the Protocol

Any changes in this research activity, except those necessary to remove an apparent, immediate hazard to the subject, must be reviewed and approved by the sponsor or its

designee. Amendments to the protocol must be submitted in writing to the investigator's IRB/IEC for approval before subjects can be enrolled into an amended protocol.

10.2.2 Protocol Deviations

The investigator or designee must document and explain in the subject's source documentation any deviation from the approved protocol. The investigator may implement a deviation from, or a change of, the protocol to eliminate an immediate hazard to study subjects without prior IRB/IEC approval. As soon as possible after such an occurrence, the implemented deviation or change, the reasons for it, and any proposed protocol amendments should be submitted to the IRB/IEC for review and approval, to the sponsor for agreement, and to the regulatory authorities, if required.

A deviation from the protocol is an unintended or unanticipated departure from the procedures or processes approved by the sponsor and the IRB/IEC and agreed to by the investigator. A significant deviation occurs when there is nonadherence to the protocol by the subject or investigator that results in a significant, additional risk to the subject. Significant deviations can include nonadherence to inclusion or exclusion criteria, enrollment of the subject without prior sponsor approval, or nonadherence to FDA regulations or ICH GCP guidelines, and will lead to the subject being withdrawn from the study (Section 4.2).

Protocol deviations will be documented by the clinical monitor throughout the course of monitoring visits. Principal investigators will be notified in writing by the monitor of deviations. The IRB/IEC should be notified of all protocol deviations in a timely manner.

10.3 Study Termination

Although Galectin Therapeutics has every intention of completing the study, Galectin Therapeutics reserves the right to discontinue the study at any time for clinical or administrative reasons.

The end of the study is defined as the date on which the last subject completes the last visit (includes follow-up visit).

10.4 Final Report

Whether the study is completed or prematurely terminated, the sponsor will ensure that the clinical study reports are prepared and provided to the regulatory agency(ies) as required by the applicable regulatory requirement(s). The sponsor will also ensure that the clinical study reports in marketing applications meet the standards of the ICH harmonised tripartite guideline E3: Structure and content of clinical study reports.

Where required by applicable regulatory requirements, an investigator signatory will be identified for the approval of the clinical study report. The investigator will be provided reasonable access to statistical tables, figures, and relevant reports and will have the opportunity to review the complete study results.

Upon completion of the clinical study report, the sponsor will provide the investigator with the full summary of the study results. The investigator is encouraged to share the summary results with the study subjects, as appropriate. The study results will be posted on publicly available clinical trial registers.

11 Schedule of Events

Table 11-1 Schedule of Events – Prescreen and Screen

**Procedure**	**Screen**
**Week**	**Week -6 to Day 0[a]**
Informed consent	X
NASH Diagnosis (Liver biopsy with NASH and Brunt stage 3 fibrosis)	X
Inclusion/exclusion criteria	X
Demographic information	X
Medical, surgical, medication history	X[b]
12-lead ECG	X
Hematology	X[c]
Blood chemistry	X[d]
Coagulation profile	X
Viral hepatitis B and C serology; HIV serology	X
Urinalysis	X[e]
Serum pregnancy test (females of childbearing potential only)	X[f]
Complete physical examination (including height [in cm] and weight [in kg] with particular attention to examination for stigmata of liver disease/cirrhosis)	X
Vital sign measurements	X[g]
MRE	X[h]
LiverMultiScan	X[h]
FibroScan	X[h]
AE monitoring	X

Abbreviations: AE = adverse event; ECG = electrocardiogram; EGD = esophagogastroduodenoscopy; IMP = investigational medicinal product; NASH = nonalcoholic steatohepatitis

a The screen visit window is up to 6 weeks. Subjects who fail to meet eligibility criteria during the screening period due to an abnormal laboratory result may undergo retesting of the abnormal laboratory parameter during the screening window and at the discretion of the investigator and with prior approval of the medical monitor.

b Medication history for the previous 3 months prior to screening should be recorded.

c Hematology will include complete blood cell count with differential (red blood cells, white blood cells, red cell indices, platelets, hemoglobin, and hematocrit)

d Blood chemistry will include alanine aminotransferase, aspartate aminotransferase, albumin, alkaline phosphatase, alpha-2 macroglobulin, bicarbonate, blood urea nitrogen, calcium, chloride, creatinine, glucose, lactate dehydrogenase, magnesium, phosphorus, potassium, sodium, total and direct bilirubin, total protein, uric acid, gamma-glutamyl transferase, fasting insulin level. Subjects must be in a fasted state prior to blood collection.

e Urinalysis will include bacteria, bilirubin, blood, crystals, color, clarity, epithelial cells, glucose, hyaline and other casts, leukocyte esterase, nitrite, pH, specific gravity, ketones, protein, RBC, WBC, mucus, and yeast.

f Serum pregnancy test for females of childbearing potential only. (A urine pregnancy test will be given every 4 weeks during the treatment phase.)

g Vital sign measurements include heart and respiratory rate, blood pressure, and body temperature. Blood pressure will be obtained with the subject in the supine position and measured twice consecutively with a 1-minute interval between measurements. The average of the 2 measurements will be recorded.

h MRE, LiverMultiScan, and Fibroscan should be conducted within 2 weeks prior to the first infusion.

Table 11-2 Schedule of Events – Randomization, Treatment Phase, and Follow-up/Early Termination

**Infusion Visit**	**1**	**2, 3, 4**	**5**	**6, 7, 8**	**9**	**7-21 days after final dose**	**30 days after final dose**
**Study Day**	**1**	**14, 28, 42**	**56**	**70, 84, 98**	**112**	**119-133**	**142**
**Visit Window (days)**		**+3**	**+3**	**+3**	**+3**	**+3**	**+3**
Limited physical examination[a]	X	X	X	X	X		X
Urine pregnancy test[b]	X		X		X		
Vital sign measurements[c]	X	X	X	X	X		X
Hematology[d]	X		X		X		
Blood chemistry[e]	X		X		X		
AE Evaluation	X	X	X	X	X		X
Concomitant Meds	X	X	X	X	X		X
IMP administration[f]	X	X	X	X	X		
12-lead ECG					X		
MRE						X	
LiverMultiScan						X	
FibroScan						X	

[a] Limited physical examination includes weight, heart, lung and abdominal examination.

[b] Urine pregnancy test will be given to females of childbearing potential at infusion 1, 5, and 9 during the treatment phase

[c] Vital sign measurements include heart and respiratory rate, blood pressure and body temperature prior to IMP administration. Blood pressure will be obtained with the subject in the supine position and measured twice consecutively with a 1-minute interval between measurements. The average of the 2 measurements will be recorded.

[d] Hematology will include complete blood cell count with differential (red blood cells, white blood cells, red cell indices, platelets, hemoglobin, and hematocrit)

[e] Blood chemistry will include alanine aminotransferase, aspartate aminotransferase, albumin, alkaline phosphatase, alpha-2 macroglobulin, bicarbonate, blood urea nitrogen, calcium, chloride, creatinine, glucose, lactate dehydrogenase, magnesium, phosphorus, potassium, sodium, total and direct bilirubin, total protein, uric acid, gamma-glutamyl transferase, fasting insulin level. Subjects must be in a fasted state prior to blood collection

[f] IMP administration will occur after the following procedures: limited physical examination, vital sign measurements, hematology, blood chemistry, and biomarker blood samples.

12 References

1. Chalasani N, Younossi Z, Lavine JE, Diehl AM, Brunt EM, Cusi K, et al. The diagnosis and management of non-alcoholic fatty liver disease: practice Guideline by the American Association for the Study of Liver Diseases, American College of Gastroenterology, and the American Gastroenterological Association. Hepatology 2012 Jun;55(6):2005-2023.

2. Williams CD, Stengel J, Asike MI, Torres DM, Shaw J, Contreras M, et al. Prevalence of nonalcoholic fatty liver disease and nonalcoholic steatohepatitis among a largely middle-aged population utilizing ultrasound and liver biopsy: a prospective study. Gastroenterology 2011 Jan;140(1):124-131.

3. Caldwell S, Argo C. The natural history of non-alcoholic fatty liver disease. Dig Dis 2010;28(1):162-168.

4. Rinella ME. Will the increased prevalence of nonalcoholic steatohepatitis (NASH) in the age of better hepatitis C virus therapy make NASH the deadlier disease? Hepatology 2011 Oct;54(4):1118-1120.

5. Di LS, Sundblad V, Cerliani JP, Guardia CM, Estrin DA, Vasta GR, et al. When galectins recognize glycans: from biochemistry to physiology and back again. Biochemistry 2011 Sep 20;50(37):7842-7857.

6. Traber PG, Zomer E. Therapy of experimental NASH and fibrosis with galectin inhibitors. PLoS One 2013;8(12):e83481.

7. Traber PG, Chou H, Zomer E, Hong F, Klyosov A, Fiel MI, et al. Regression of fibrosis and reversal of cirrhosis in rats by galectin inhibitors in thioacetamide-induced liver disease. PLoS One 2013;8(10):e75361.

8. Kim D, Kim WR, Talwalkar JA, Kim HJ, Ehman RL. Advanced fibrosis in nonalcoholic fatty liver disease: noninvasive assessment with MR elastography. Radiology 2013 Aug;268(2):411-419.

9. Banerjee R, Pavlides M, Tunnicliffe EM, Piechnik SK, Sarania N, Philips R, et al. Multiparametric magnetic resonance for the non-invasive diagnosis of liver disease. J Hepatol 2014 Jan;60(1):69-77.

10. Wong VW, Vergniol J, Wong GL, Foucher J, Chan HL, Le BB, et al. Diagnosis of fibrosis and cirrhosis using liver stiffness measurement in nonalcoholic fatty liver disease. Hepatology 2010 Feb;51(2):454-462.

13 Protocol Amendments

This section provides a summary of significant changes implemented via protocol amendment to the previous version of the protocol. Minor changes that do not affect the material substance or intent of the original protocol, such as correction of grammatical/typographical errors, minor wording/editorial revisions, date revisions, format changes, etc. are not described in the Description of Changes Table.

13.1 Amendment 1

Location of Change	Description of Change and Rationale (if needed)
• Protocol Synopsis<br>• 2.3 Exploratory Objectives<br>• 3.1 Study Design<br>• 6.1.2.3 Infusion Visits<br>• 7.3 Exploratory Endpoints<br>• 11 Schedule of Events	Removed collection of additional serum for assessment of an experimental set of novel biomarkers developed by Dr. Harrison.<br>**Rationale:** Requested by Brooke Army Medical Center IRB.
• Protocol Synopsis<br>• 3.1 Study Design<br>• 4.1.1 Inclusion Criteria<br>• 6.1.1 Screening Visit<br>• 11 Schedule of Events	Removed Brunt Stage 4 fibrosis.<br>**Rationale:** Study is evaluating patients with advanced bridging fibrosis, not cirrhosis.
• 3.1 Study Design	Clarified that concomitant medications are assessed at each visit & a urine pregnancy test is done at the 1st, 5th, and 9th infusion visits.
• 4.1.2 Exclusion Criteria<br>• 5.9 Prohibited Medications or	Removed exclusion of drugs with a narrow therapeutic window metabolized by CYP3A4 including specifically the fast acting opioids alfentanil and fentanyl, the immunosuppressive drugs cyclosporine, sirolimus, and tacrolimus, the cardiovascular agents

Location of Change	Description of Change and Rationale (if needed)
Therapies	ergotamine, quinidine and dihydroergotamine, and the psychotropic agent pimozide.<br>**Rationale:** Results of GT-029 Drug-Drug Interaction Study performed to evaluate CYP3A4 enzyme activity utilizing midazolam confirmed that there were no drug-drug interactions and no serious adverse events or drug-related adverse events.
• 4.1.2 Exclusion Criteria	Added "Any subject who cannot undergo an MRI, e.g., due to certain metal or electronic device implants, as determined by the Principal Investigator."<br>**Rationale:** Requested by Brooke Army Medical Center IRB
• 11 Schedule of Events	Added footnote to Table 11-1 to emphasize that MRE, LiverMultiScan, and Fibroscan should be conducted within 2 weeks prior to the first infusion.<br>Removed "Screen" and "0" columns from Table 11-2 as they are redundant to Table 11-1.<br>Added Study Day and Visit Window header rows to Table 11-2 to clarify the time relationship to the infusion visits.<br>Removed urine pregnancy test collection from 30-day follow-up visit in Table 11-2 as this is only performed at infusion visits 1, 5, and 9.

13.2 Amendment 2

Location of Change	Description of Change and Rationale (if needed)
• Protocol Synopsis<br>• 3.1 Study Design<br>• 4.1.1 Inclusion Criteria	Changed requirement for a liver biopsy confirmed diagnosis of NASH from within *6 months* of randomization to within *12 months* of randomization.<br>**Rationale**: Reduced the patient burden of undergoing a repeat of an invasive procedure more frequently than necessary when a liver biopsy result within the past year is adequate to document the NASH Brunt stage 3 diagnosis eligibility criterion.
• Protocol Synopsis<br>• 2.3 Exploratory Objectives<br>• 3.1 Study Design<br>• 6.1.1 Screening Visit<br>• 6.1.2.3 Infusion Visits<br>• 6.3.8 Blood Chemistry<br>• 6.4 Sample Collections<br>• 7.3 Exploratory Endpoints<br>• 11 Schedule of Events	Serum will also be analyzed for alpha-2 macroglobulin.<br>**Rationale:** Inadvertently omitted from original protocol. An additional blood draw will *not* be required.
• 5.2 Identity of Investigational Product	Added the following sentence: *"The dose calculated based on the LBM at the first infusion visit will be maintained at each subsequent infusion visit since it is highly unlikely that LBM will vary significantly (e.g. by 10%) during the course of the study."*<br>**Rationale:** To clarify that the study medication dose administered at the first infusion visit, as determined by LBM, will not change during the study and will remain the same at each subsequent infusion visit.

Location of Change	Description of Change and Rationale (if needed)
• 5.8 Prior and Concomitant Therapy	Deleted the sentence stating that if prohibited medications are needed for the welfare of the subject, the subject should be discontinued from the study.<br>**Rationale:** There are no prohibited medications in this study.

13.3 Amendment 3

Location of Change	Description of Change and Rationale (if needed)
• 4.1.2 Exclusion Criteria	Added the following bolded phrase to Exclusion Criterion #12:<br>Any history of malignancy ***in the previous 5 years in the judgement of the Principal Investigator***, except for the following adequately-treated non metastatic basal cell skin cancer; any other type of skin cancer, except melanoma, that has been adequately treated and has not recurred for at least 1 year prior to enrollment; and adequately treated in situ cervical cancer that has not recurred for at least 1 year prior to enrollment.<br>**Rationale**: Mistakenly omitted in the original protocol. To allow consideration of subjects with a remote history of cancer that is deemed cured with a low chance of recurrence, in the judgement of the Principal Investigator.
• 6.3 Safety Assessments	Removed verbiage regarding evaluation of immunogenicity of GR-MD-02.<br>**Rationale**: Verbiage erroneously included in original protocol; immunogenicity of GR-MD-02 will not be evaluated in this clinical study.
